# Biodegradable Piezoelectric Micro‐ and Nanomaterials for Regenerative Medicine, Targeted Therapy, and Microrobotics

**DOI:** 10.1002/smsc.202400439

**Published:** 2025-01-28

**Authors:** Lorenzo Vannozzi, Carlotta Pucci, Diego Trucco, Claudia Turini, Semih Sevim, Salvador Pané, Leonardo Ricotti

**Affiliations:** ^1^ The BioRobotics Institute Scuola Superiore Sant’Anna Piazza Martiri della Libertà 33 56127 Pisa Italy; ^2^ Department of Excellence in Robotics & AI Scuola Superiore Sant’Anna Piazza Martiri della Libertà 33 56127 Pisa Italy; ^3^ Multi‐Scale Robotics Lab (MSRL) Institute of Robotics and Intelligent Systems (IRIS) ETH Zurich 8092 Zurich Switzerland

**Keywords:** biodegradation, minimally invasive therapy, nanorobots, organic nanomaterials, piezoelectricity, regenerative medicine

## Abstract

Piezoelectric micro‐ and nanomaterials can generate local electrical signals when subjected to mechanical stress, a phenomenon that can be exploited to trigger beneficial effects at the cell and tissue level. In recent years, research on biodegradable piezoelectric material has gained momentum, as these materials can degrade after fulfilling their function. Thus, they promise to considerably impact regenerative medicine, targeted therapy, and microrobotics, with better chances to match regulatory requirements with respect to their nondegradable counterparts. This review offers a comprehensive overview of recent advancements in biodegradable piezoelectric micro‐ and nanomaterials, focusing on their piezoelectric mechanisms, material types, and methods to enhance their properties. Current characterization techniques, emphasizing both piezoelectricity and biodegradability at the micro/nano scale, are also discussed. Furthermore, it is discussed how to use these materials in intelligent platforms for regenerative medicine and responsive drug delivery systems. The application of piezoelectric micro‐ and nanomaterials in microrobotics is also examined, particularly their potential for minimally invasive procedures. Finally, challenges and future directions are highlighted, underscoring the importance of biodegradable piezoelectric materials as versatile platforms for advancing biomedical technologies.

## Graphical abstract

This article outlines the key efforts in the state‐of‐the‐art of biodegradable piezoelectric nanomaterials and their possible impact in the biomedical field. Material types and compositions, fabrication techniques, methods to enhance piezoelectric properties, and characterization approaches are described. Specific applications of these materials in regenerative medicine, targeted therapy, and microrobotics are discussed, highlighting current challenges and future directions.

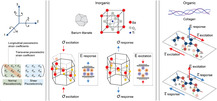

## 1 Introduction

Micro‐ and nanomaterials are particularly attractive due to their high surface energy, targeting properties, and cell–material interactions. The dominance of surface forces over volume forces at small scales underscores the relevant role assumed by micro‐ and nanomaterials and their potential in reshaping the landscape of various scientific disciplines, from materials science to medicine and beyond. The last decade has seen, in particular, remarkable advancements in the field of piezoelectric materials.

Piezoelectricity is an intrinsic property of certain materials, which can convert a mechanical response into electrical charges (direct piezoelectricity) and vice versa (converse piezoelectricity).^[^ ^1^ ^]^ The unique characteristics of piezoelectric inorganic materials ground on their nonsymmetric crystal structure, which allows the alignment of opposite ions to form an electric dipole moment. Dipoles are uniformly distributed and oriented throughout the crystal regions of the material, forming domain structures. These domains can be aligned in a single direction to uniformize the material polarization, thus increasing the piezoelectric response.

**Figure 1:**
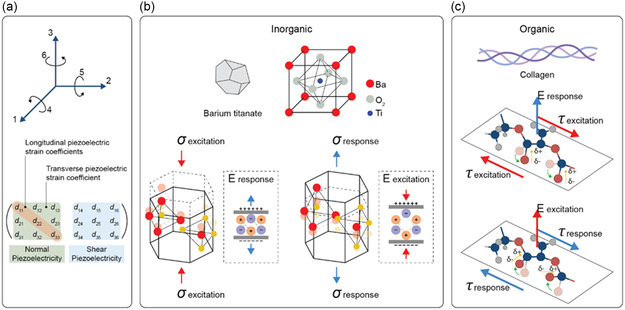
a) Schematic representation of the directions in the Voigt notation, and the piezoelectric tensor describing the coefficient responsible for the normal and shear piezoelectricity. Chemical formulas and crystal structures shown as examples of b) an inorganic (e.g., barium titanate) and c) organic (e.g., collagen) material. Inorganic materials are featured by a higher piezoelectric response in the same direction as the applied stimulus, while organic ones often show a higher shear behavior.

The piezoelectricity depends on the material composition, including the orientation and symmetry of the crystals. The applied stress (*T*) and the resulting electric polarization (*P*) in a piezoelectric material are proportionally related as follows:

(1) P=d×T

where *d* corresponds to the piezoelectric coefficient tensor, which describes the piezoelectric activity of the material. This coefficient can be expressed as a third‐rank tensor with 3 × 6 components indicated as *d*_*ij*_, where the subscript *i* represents the directions of the generated polarization and *j* represents the direction of the applied stress. The subscripts from 1 to 3 denote the X, Y, and Z directional axes, whereas the subscripts from 4 to 6 indicate the shear planes that are perpendicular to each of those axes, respectively (**Figure** **1**a). Piezoelectricity is a direction‐dependent property, as each component is defined with a magnitude and a direction, positive or negative, depending on the resulting strain or induced polarization concerning their reference axis.^[^ ^2^ ^]^

Piezoelectricity is often found in natural structures, including human body tissues. Since the discovery of piezoelectric polarization in wool and hair in 1941,^[^ ^3^ ^]^ piezoelectricity has been observed in various human tissues, including bone, tendon, and skin.^[^ ^4^ ^]^ At a smaller scale, piezoelectricity has been observed in proteins, amino acids, and polysaccharides, wherein the physical principles of piezoelectricity varied with respect to inorganic materials. Indeed, organic materials possess well‐organized and highly ordered structures that exhibit low symmetry and lack an inversion center. Despite that, the functional groups and the spatial configurations of organic polymers lead to unconventional sources of piezoelectricity.^[^ ^5^ ^]^ For instance, the piezoelectric properties of macromolecules such as long‐chain peptides, proteins, polysaccharides, and synthetic polymers are influenced not only by intramolecular dipoles but also by other factors such as hydrogen‐bond networks, spatial folding, and helical and fibrous structures.^[^ ^6^ ^]^

Piezoelectric materials offer several advantages over conventional biomaterials, as they can be used to transmit electricity to living systems in response to processes, such as body movements or external stimulation (e.g., ultrasound).^[^ ^7^ ^]^ For instance, piezoelectricity has shown healing potential for hard and soft tissues. Indeed, when a tissue such as bone or cartilage is compressed or, in some other cases, when a tendon, ligament, or skin is stretched, electrical stimuli are generated.^[^ ^8^ ^]^ For example, the human tibia can generate up to 300 μV of piezoelectric potential during walking.^[^ ^9^, ^10^ ^]^ According to Wolff's law, bone is susceptible to remodeling when subjected to stress, and collagen piezoelectricity has been invoked as one of the main potential mechanisms behind this process.^[^ ^11^ ^]^ Therefore, mechanically induced electrical energy has the potential to exert a significant regulatory effect at both cellular and molecular levels, for example in the activation of cell pathways (e.g., calcium signaling pathways) and for the regulation of mechanosensitive channels. In the former case, piezoelectric materials can induce localized electric fields that lead to the opening of voltage‐gated calcium channels on the cell membrane that may regulate important activities in eukaryotic cells, as depolarization signals in neurons, and contribute to synaptic activity.^[^ ^10^, ^12^ ^]^ In the latter case, the PIEZO1 and PIEZO2 protein receptors are among the most extensively studied mechanosensitive transducers in cells and enable the detection of cell membrane deformation due to mechanical stimuli.^[^ ^13^, ^14^ ^]^ The activation of these channels contributes to the regulation of various cellular activities, including osmoregulation, volume control, and cellular responses to mechanical forces.

Piezoelectricity, like ferroelectricity, concerns the electroresponsive behavior of certain materials, but they have distinct characteristics and underlying mechanisms. Ferroelectricity is a property of certain materials that exhibit a spontaneous electric polarization (a permanent dipole moment) that can be reoriented by applying an external electric field. In other words, the spontaneous polarization in ferroelectric materials naturally leads to a piezoelectric response when the material is mechanically deformed. All ferroelectric materials are piezoelectric; however, not all of them are ferroelectric.

Compared to their bulk counterparts, piezoelectric micro‐ and nanomaterials possess outstanding advantages in eliciting cell‐specific responses. This is attributed to their size, and shape (flakes, wires, rods, fibers, tubes, particles, film), thus in their high surface‐area‐to‐volume ratio, which results in high surface energy, as well as the potential presence of nanotopography and its impact on surface energy.^[^ ^15^ ^]^ It is worth remarking that miniaturizing piezoelectric materials to the micro‐ and nanoscale has significant consequences. Indeed, micro‐ and nanoscale piezoelectric structures can exhibit an enhancement or decrease in their piezoelectric coefficients compared to their macroscale counterparts owing to various factors, including the concentration and elimination of crystal surface defects or the contraction or expansion of the crystal lattice.^[^ ^16^ ^]^ However, downscaling allows a faster response with a reduced need for the voltage, or mechanical magnitude to impose a deformation, required for performing a specific electromechanical task.^[^ ^17^ ^]^ In theory, the driving energy for piezoelectric nanostructures can be substantially less than the one needed to operate macroscale piezoelectric transducers.

Piezoelectric micromaterials are usually used in applications requiring larger forces or displacements, such as in ultrasonic transducers or microactuators. On the contrary, nanomaterials are more suitable for applications requiring high sensitivity, low power, or incorporation into nanoscale devices, such as biomedical sensors, energy harvesters, or wearable electronics. Indeed, piezoelectric nanotransducers, lighter than bulk materials, are also more suitable for integration on flexible substrates, which may better conform to tissues when implanted than more rigid piezoelectric bulk materials. Piezoelectric micro‐ and nanomaterials have been exploited for various applications in the fields of regenerative medicine,^[^ ^18^, ^19^ ^]^ targeted therapy and controlled drug delivery,^[^ ^20^ ^]^ and microrobotics.^[^ ^21^ ^]^ In regenerative medicine, the primary tissue targets include bone, skin, nerve, cardiac, and muscle tissues, showing promising outcomes such as improved bone healing and integration, accelerated nerve cell differentiation and neural network formation, support for the regeneration of damaged myocardial tissue, and enhanced myoblast differentiation.^[^ ^22^ ^]^

For targeted therapy and microrobotics, the focus has primarily been on developing responsive drug delivery systems and cancer treatments, enabling precise stimulation and delivery with high spatial accuracy. Piezoelectric nanomaterials can also be easily injected into tissues, inhaled, or released into circulation and used to elicit drug release by applying external ultrasound stimulation,^[^ ^19^ ^]^ or in conjunction with other materials such as magnetic, optical, and plasmonic.^[^ ^23^ ^]^ Nonetheless, these piezoelectric materials must be biocompatible and possibly biodegradable so they can be safely applied to biological systems and interfaced with biological tissues.^[^ ^24^ ^]^

In all these applications, piezoelectric nanomaterials show clear advantages over competing technologies, such as implanted electrodes for electrical stimulation of deep tissues, as they avoid the need for invasive procedures and allow a specific and targeted action, up to the single cell level.

Several materials have been examined for various uses, such as sensing, harvesting, actuation, and other biomedical devices. Lead zirconate titanate (PZT), potassium sodium niobate (KNN), barium titanate (BTO), and lithium niobate (LiNbO_3_) have been widely recognized as inorganic materials with excellent piezoelectric properties.^[^ ^25^ ^]^ Regardless, their nondegradability and potential leakage of hazardous or toxic constituents pose challenges in their use for biomedical implants today. Since 2014, the Restriction of Hazardous Substances (RoHS) Directive (2011/65/EU, also known as RoHS II) has been applied to medical devices standardizing the use of hazardous materials and, therefore, limiting the presence of toxic elements. In particular, lead‐based materials such as the PZT have raised issues due to the brittleness of these ceramics and the toxicity of Pb released from the implant.^[^ ^26^ ^]^ In addition, risks of infection or potential material rejection constitute other possible unsafe consequences of the implantation, which may require a second intervention for material retrieval. Using coatings or encapsulating materials has been suggested to mitigate potential toxic concerns,^[^ ^27^ ^]^ although optimal solutions are still under investigation. Apart from the intrinsic toxicity of the material, the accumulation of nondegradable piezoelectric particles may also lead to harmful consequences in the long term, especially when gathered in targeted organs via blood circulation over time. In this case, in vivo fate and biodistribution undoubtedly vary based on the interplay of the size, surface charge, and chemical composition, leading to potential unexpected concerns.^[^ ^28^ ^]^

In terms of dimensions, it has been recognized that large rigid particles with a diameter >2 μm preferentially accumulate within the spleen, liver, and lungs, nanoparticles with a range of 20–150 nm may partially escape filtration by the liver and spleen, while only small‐sized nanoparticles or particle fragments (hydrodynamic diameter < 5.5 nm) are cleared by the kidneys.^[^ ^29^ ^]^ Accumulation of inorganic material may lead to chronic inflammation, as the persistent presence of nondegradable particles can cause ongoing inflammation of organs and tissue damage, and the repeated activation of the immune system may lead to hypersensitivity and autoimmune responses. It has been observed that long‐term exposure to nanomaterials is associated with increased levels of inflammatory mediators, such as tumour necrosis factor (TNF‐α) and Cxcl2, as well as an increased risk of fibrosis.^[^ ^30^ ^]^

Monitoring the long‐term safety and efficacy of products involving particles is of primary importance. However, this approach requires a longitudinal study and the use of markers in conjunction with appropriate imaging systems to evaluate the particle fate over time.

Biodegradable piezoelectric materials have attracted a growing interest in recent years. They are capable of safely dissolving in the host environment (e.g., bodily fluids), in a hydrolytic or enzymatic degradation process that does not produce any harmful byproducts, circumventing the need for additional surgical procedures.^[^ ^31^ ^]^ According to their nature, biodegradable piezoelectric polymers can be categorized into synthetic polymers (such as poly‐L‐lactic acid), and natural polymers, as proteins (such as collagen and silk), polysaccharides (such as cellulose, chitin, and chitosan), peptides/amino acids, and other compounds (e.g., virus).

Numerous reviews have been devoted to piezoelectric biomaterials with a particular emphasis on different types of biomaterials or specific applications, such as energy harvesting, sensing, and actuation.^[^ ^32^, ^33^, ^34^ ^]^ Several reviews have reported the perspectives of utilizing biological piezoelectric polymers.^[^ ^35^, ^36^, ^37^, ^38^ ^]^ Nonetheless, none of these works have explored the potential of degradable piezoelectric micro‐ and nanomaterials, which may hold great potential in targeting therapies at the micro‐ and nanoscale levels.

This review provides a comprehensive overview of the research ongoing in the field of piezoelectric and biodegradable micro‐ and nanomaterials, with a particular emphasis on their applications in regenerative medicine, targeted therapy, and microrobotics. We classify these piezoelectric materials into distinct groups based on their inherent characteristics. We also discuss the strategies for their synthesis, analysis, and improvement of piezoelectric performance. An overview of the biodegradation mechanism of organic piezoelectric materials is provided, with a particular emphasis on the intracellular pathways activated by biodegradable nanomaterials. The application of such piezoelectric biomaterials in regenerative medicine, targeted therapy, and microrobotics is discussed, highlighting possible future perspectives.

## 2 Synthesis and Characterization of Biodegradable Piezoelectric Nanomaterials

### 2.1 Materials and Techniques to Improve Piezoelectricity

#### 2.1.1 Materials

In inorganic materials, the crystalline structure determines the piezoelectric properties; materials exhibiting a noncentrosymmetric geometry can produce electrical dipoles upon mechanical perturbation. The direction and magnitude of the generated electric charge are contingent upon the material's crystallographic alignment and the applied mechanical stress (Figure 1b). Although inorganic crystals, such as quartz and Rochelle salt, are the most known piezoelectric materials,^[^ ^2^ ^]^ inorganic ceramics like PZT, BTO, KNN, ZnO, boron nitride, and LiNbO_3_ have been shown to possess good piezoelectric properties (**Table** **1**). Despite their notable performance, the application of inorganic piezoelectric materials in medicine may encounter challenges concerning their biocompatibility and biodegradability. For example, lead‐based piezoelectric materials possess an inherent toxicity due to the presence of the lead element.^[^ ^39^ ^]^ To this aim, the in vivo biocompatibility of lead‐free piezoelectric materials (BTO, KNN, LiNbO3) has been investigated;^[^ ^40^ ^]^ however, their degradation products (e.g., Nb) can cause toxicity, as demonstrated in mice and rat models.^[^ ^41^ ^]^

**Table 1:** Comparison of different piezoelectric materials in terms of type, structure, synthesis approach for the fabrication of micro‐ and nanomaterials, and piezoelectric coefficients.

Type	Material	Structure/mechanism of piezoelectricity	Synthesis	Piezoelectric coefficients	References
Inorganic	Lead zirconate titanate (PZT)	Perovskite, ferroelectric	Aqueous precipitation method, hydrothermal synthesis	*d*_31_ = 93.5 to −274 pC N^−1^ *d*_33_ = 225–590 pC N^−1^	[19, 226]
	Barium titanate (BaTiO_3_, BTO)	Perovskite, ferroelectric	Hydrothermal synthesis	*d*_31_ = −33.4 to −78 pC N^−1^ *d*_33_ = 90–788 pC N^−1^	[19]
	Potassium sodium niobate (KNN)	Perovskite, ferroelectric	Solid‐state process	*d*_33_ = 93–700 pC N^−1^	[227]
	Lithium niobate (LiNbO_3_)	Perovskite, ferroelectric	Sol‐gel method, Solid‐state process, hydrothermal synthesis	*d*_31_ = −1 pC N^−1^ *d*_33_ = 16–41.5 pC N^−1^	[1, 228]
	Boron nitride	Wurtzite, nonferroelectric	Arc discharge method, chemical vapor deposition	*d*_11_ = 0.5–1.27 pC N^−1^ *d*_33_ = 0.3 pC N^−1^	[229]
	Zinc oxide (ZnO)	Wurtzite, nonferroelectric	Vapor–solid growth process	*d*_31_ = −5 pC N^−1^ *d*_33_ = 6–13 pC N^−1^	[19, 230]
Synthetic polymers	PVDF	Orthorhombic β‐phase crystal, ferroelectric	Electrospinning, solvent casting	*d*_31_ = 23 pC N^−1^ *d*_33_ = −24–34 pC N^−1^	[44]
	Polyvinylidene fluoride ‐ trifluoroethylene (PVDF‐TrFE)	β‐crystalline form, ferroelectric	Electrospinning, Solvent casting	*d*_33_ = 30 pC N^−1^	[42, 43]
	Nylon‐11	δ′‐phase crystalline structure, ferroelectric	Electrospinning, antisolvent method	*d*_31_ = 14 pC N^−1^ *d*_33_ = 3.8–4 pC N^−1^	[19, 231]
	Poly‐L‐lactic acid (PLLA)	β‐crystalline form, nonferroelectric	Melt‐press template wetting method, electrospinning, solvent casting	*d*_14 _≈ 3.7–20 pC N^−1^ *d*_31_ = 1.58 pC N^−1^ *d*_33_ = 3.1 pC N^−1^	[19, 47, 51, 232]
	Polyhydroxybutyrate (PHB)	Orthorhombic crystal, ferroelectric	Electrospinning, dip coating	*d*_14_ = 1–2 pC N^−1^ *d*_31_ = 3.25 pC N^−1^ *d*_33_ = 2.1–4.13 pC N^−1^	[35, 50, 233]
Natural‐derived polymers	γ‐Glycine	Trigonal crystal systems of piezoelectric γ‐phase structure, nonferroelectric	Solvent evaporation	*d*_31_ = 3.2 pC N^−1^ *d*_33_ = 4.1–10.4 pC N^−1^	[62, 185, 186]
	β‐Glycine	Monoclinic crystal systems of piezoelectric β‐phase structure, nonferroelectric	Solvent casting/evaporation	*d*_16_ = 178 pm V^−1^ *d*_22_ = −5.7 pm V^−1^ *d*_33_ = 4.7 pC N^−1^	[61, 212]
	Poly‐γ‐methyl‐L‐glutamate (PMLG)	Crystals of piezoelectric β‐phase structure, nonferroelectric	Electrospinning, electrospray	*d*_14_ = −2 pC N^−1^	[234, 235]
	Poly‐γ‐benzyl‐L‐glutamate (PBLG)	α‐helical peptide, nonferroelectric	Electrospinning, electrospray	*d*_33_ = 15–25 pC N^−1^	[53]
	Diphenylalanine (FF)	Noncentrosymmetric hexagonal crystal structure, nonferroelectric	Self‐assembly	*d*_14_ = −10 pC N^−1^ *d*_15_ = 80 N^−1^ *d*_33_ = 9.9–17.9 pm V^−1^	[63, 64, 65, 236]
	Collagen	α‐helixes wrapped in right‐handed fashion, nonferroelectric	Self‐assembly	*d*_14_ = −0.2–12 pm V^−1^ *d*_15_ = 1 pC N^−1^ *d*_33_ = 4.8 pm V^−1^ *d*_35_ = 27 pm V^−1^	[54, 73, 75, 237]
	Chitin	Polysaccharides founded in three forms in nature (α, β, and γ), nonferroelectric	Self‐assembly	*d*_14_ = −0.2–1.5 pC N^−1^ *d*_33_ = 9.49 pC N^−1^	[54, 77]
	Chitosan	Noncentrosymmetric orthorhombic structure, nonferroelectric	Postcasting neutralization, solvent casting	*d*_31_ = 6–10 pC N^−1^ *d*_33_ = 0.8–18.4 pC N^−1^	[79]
	Keratin	α‐helical peptide, nonferroelectric	Self‐assembly	*d*_14_ = −0.1 to 1.8 pC N^−1^	[238]
	Silk	‐sheet crystallinity, nonferroelectric	Self‐assembly	*d*_14_ = −1.5 pC N^−1^	[83]
	Cellulose	Crystalline (cellulose I) and less ordered cellulose domains, nonferroelectric	Pressure filtering	*d*_14_ = −0.1–0.2 pC N^−1^ *d*_33_ = 4.7–82.6 pC N^−1^	[69]
	M13 bacteriophage	Right‐handed helical structures, nonferroelectric	Enforced infiltration	*d*_33_ = 7.8 pm V^−1^	[84]

Piezoelectric polymers have emerged as an alternative and promising solution to inorganic materials. Among the various options, polyvinylidene fluoride (PVDF) and its copolymer, as the poly (vinylidene fluoride*‐co*‐trifluoroethylene) (PVDF‐TrFE), stand out as the most frequently exploited piezoelectric polymers.^[^ ^42^ ^]^ The reported *d*_33_ for PVDF‐TrFe is around 30 pC N^−1^,^[^ ^43^ ^]^ which is significantly lower than that observed in inorganic materials (e.g., KNN has a *d*_33_ between 93 and 700 pC N^−1^, depending on the processing conditions^[^ ^19^ ^]^). PVDF has good biocompatibility; however, it is not degradable in biological environments, limiting its applications in the medical field.^[^ ^44^ ^]^

The use of piezoelectric materials featuring both biocompatibility and biodegradability would greatly enlarge the spectrum of applications in biomedicine. Also, one of the peculiar aspects of organic materials is their noticeable shear piezoelectricity generated from the alignment and deformation of molecular dipoles within the material's structure (Figure 1c). In general, the majority of biodegradable piezoelectric materials exhibit a robust shear piezoelectricity, but show weak transverse and longitudinal piezoelectric properties. Despite their generally lower piezoelectric response compared to inorganic counterparts, the advantages conferred by their biocompatible and biodegradable nature may outweigh this limitation.^[^ ^36^ ^]^

Several examples of biodegradable piezoelectric materials derive from various sources, with different structures and degradation pathways. Generally, these materials can be divided into synthetic polymers and natural piezoelectric ones.

The former include poly lactic acid (PLA) and poly‐L‐lactic acid (PLLA, a conformation of PLA containing only the L‐stereoisomer), polyhydroxybutyrate (PHB), poly‐γ‐benzyl‐L‐glutamate (PBLG), or poly‐γ‐methyl‐L‐glutamate (PMLG). PLA is synthesized by polycondensation of lactic acid (LA) monomers, or by ring‐opening polymerization of lactides.^[^ ^45^ ^]^ To obtain the pure L‐stereoisomer (PLLA), the synthesis should start from the optically pure L‐LA isomer; this is attained only when LA is synthesized by microbial fermentation. The piezoelectric properties observed in PLA and PLLA derive from their semicrystalline structure, characterized by a chiral conformation and the alignment of electric dipoles in the C=O groups along the polymer backbone. In the thermodynamically stable α‐form of PLA, the C=O groups exhibit random orientation, resulting in negligible piezoelectric effects. Conversely, in the β‐crystalline form, the C=O groups align along the main chain, imparting piezoelectric properties to the material.^[^ ^46^ ^]^ Achieving this orientation in PLA and PLLA necessitates specific processing techniques, such as thermal stretching or electrospinning, which promote the rearrangement of molecular chains along the stretching direction. PLLA exhibits piezoelectricity predominantly along the Z‐axis, characterized by a *d*_14_ coefficient typically ranging from 9 to 11 pC N^−1^,^[^ ^47^ ^]^ which is notably lower than inorganic piezoelectric materials. Nevertheless, PLLA continues to be extensively utilized in various biomedical applications owing to its biocompatibility and biodegradability. Furthermore, post‐treatment procedures aimed at enhancing its molecular orientation and crystallinity can potentially increase its piezoelectric performance (*d*_14_ coefficient) up to 20 pC N^−1^. The piezoelectric properties of PLLA also depend on its optical purity and molecular weight. Tajitsu demonstrated that PLLA with a lower D‐isomer content and high molecular weight might result in films with a higher piezoelectric response.^[^ ^48^ ^]^ Similarly, Schönlein et al. showed that by increasing the D‐content from <1% up to 12%, the *d*_14_ decreases from 3.7 ± 0.3 pC N^−1^ to 0.^[^ ^49^ ^]^

PHB is a biodegradable polyester that belongs to the class of polyhydroxyalkanoates (PHAs), naturally occurring biopolymers synthesized by microorganisms under conditions of nutrient limitation.^[^ ^50^ ^]^ The piezoelectric features of PHA‐based materials stem from the presence of an asymmetric carbon atom linked to a polar oxygen group.^[^ ^51^ ^]^ In the case of pure PHB electrospun nanofibers, the piezoelectric coefficients are ≈3.25 pC N^−1^ (*d*_31_) and 4.13 pC N^−1^ (*d*_33_).^[^ ^50^ ^]^ The molecular weight of PHB impacts its piezoelectric properties; in fact, it has been observed that a decrease in the molecular weight from 803 to 102 kDa resulted in a lower number of piezoelectric domains in PHB films, leading to a lower piezoresponse.^[^ ^52^ ^]^

PBLG is a synthetic polypeptide derived from the polymerization of γ‐benzyl‐L‐glutamate. It has a repeating unit of glutamic acid with a benzyl ester side chain. The structure of PBLG allows it to form helical conformations, contributing to its unique piezoelectric properties. Indeed, PBLG exhibits piezoelectric properties primarily due to its ordered helical structure, which lacks inversion symmetry, an essential requirement for piezoelectricity. This material has been studied for its interesting mechanical, structural, and piezoelectric properties. PBLG showed a *d*_33_ coefficient in the form of fibers up to 25 pC N^−1^.^[^ ^53^ ^]^

PMLG is a synthetic biopolymer obtained by polymerization of γ‐methyl‐L‐glutamate repeating units. Belonging to the broader class of polyamides, PMLG is characterized by the presence of amide bonds (–CONH–) in its polymer backbone. PMLG shows a relatively low shear piezoelectricity (*d*_14_ = −2 pC N^−1^) with respect to the previous synthetic polymers.^[^ ^54^ ^]^

The exploration of natural piezoelectric materials garnered significant attention due to their implications in vital physiological processes, thereby driving extensive research into their piezoelectric properties and potential medical applications.^[^ ^36^ ^]^ For instance, it has been demonstrated that bone tissue displays piezoelectric behavior, and this peculiar activity is responsible for crucial functions such as bone remodeling and healing.^[^ ^22^ ^]^ Furthermore, other naturally occurring macro materials, such as spider silk,^[^ ^55^ ^]^ fish collagen,^[^ ^56^, ^57^ ^]^ chicken feathers,^[^ ^58^ ^]^ prawn shells,^[^ ^59^ ^]^ and porcine skin,^[^ ^60^ ^]^ have demonstrated varying degrees of piezoelectricity.

The precise origins of piezoelectricity in polymers remain subject to ongoing investigations; nonetheless, it has been observed that many biological materials adopt helical or spiral conformations, lacking a center of symmetry, thereby endowing them with piezoelectric capabilities.^[^ ^22^ ^]^

Among the natural piezoelectric materials, amino acids and peptides (the fundamental constituents of biological materials) have garnered attention. With 19 out of the 20 natural amino acids exhibiting chirality and thus lacking crystal symmetry,^[^ ^61^ ^]^ experimental studies have confirmed their piezoelectricity, except for methionine.^[^ ^2^ ^]^ The only nonchiral amino acid, glycine, exhibits crystallization in three distinct polymorphs: α, β, and γ. The α‐form, characterized by an antiparallel molecular arrangement, does not manifest piezoelectric behavior due to the cancellation of net polarization.^[^ ^62^ ^]^ Conversely, both the γ and β forms display piezoelectricity owing to their noncentrosymmetric geometries. However, the β‐form is metastable and primarily observed in nanoconfined environments; under typical ambient conditions, it tends to revert to the more stable γ‐form. Measurements reveal that γ‐glycine possesses a longitudinal piezoelectric coefficient *d*_33_ of ≈10 pm V^−1^,^[^ ^62^ ^]^ whereas β‐glycine exhibits a lower longitudinal coefficient *d*_22_ of ≈−5.7 pm V^−1^, alongside a notably high shear piezoelectric coefficient *d*_16_ of around 178 pm V^−1^.^[^ ^61^ ^]^

Diphenylalanine (FF), a peptide composed of two phenylalanine units, has also generated attention due to its piezoelectric and mechanical properties. Due to its peculiar structure, FF can self‐assemble into a tubular supramolecular structure through hydrogen bonding and *π*–*π* stacking interactions.^[^ ^63^ ^]^ FF nanotubes with hexagonal (P_6_) crystal structure exhibit high shear piezoelectricity (*d*_15_) along the tube axis. Furthermore, studies have revealed that the orthorhombic form of FF nano‐ and microtubes also possesses shear piezoelectricity. Some authors also measured longitudinal coefficients *d*_33_ between 9.9 and 17.9 pm V^−1^.^[^ ^64^, ^65^ ^]^ It is worth mentioning that the piezoelectric behavior of FF nanotubes is contingent upon their unidirectional growth and dimensions.

Cellulose, a linear polysaccharide comprised of glucose units, possesses a fibrous crystalline arrangement, imparting mild piezoelectric characteristics.^[^ ^2^ ^]^ Cellulose is the primary structural constituent of plant cell walls and thus contributes to plant rigidity, strength, and overall structural integrity.^[^ ^66^ ^]^ Cellulose chains have a net dipole; however, in plants, cellulose microfibrils follow an antiparallel alignment, thus resulting in a net cancellation of the dipole moment at the macroscopic scale.^[^ ^2^ ^]^ This linear configuration facilitates extensive hydrogen bonding between adjacent cellulose chains, contributing to its remarkable strength and insolubility in water. At the micro‐ and nanoscale, cellulose crystals display piezoelectric properties due to their alignment in both positive and negative orientations along the Z‐direction, while maintaining a random orientation in the X–Y plane.^[^ ^67^ ^]^ Cellulose derived from wood exists in two polymorphs: a triclinic crystal structure with no symmetry, (*I*_α_) or a monoclinic crystal structure (*I*_β_). The presence of hydrogen bonds, particularly prominent in the 2D *I*_β_ polymorph, significantly contributes to cellulose piezoelectric properties. Various cellulosic materials, including cellulose nanocrystals, cellulose nanofibers, and bacterial nanocellulose sourced from onion skin and bleached birch cellulose, have piezoelectric behavior, albeit with low magnitudes (<1 pC N^−1^).^[^ ^68^ ^]^ Rajala et al. demonstrated that films composed of cellulose nanofibrils with monoclinic symmetry (space group *C*_2_∥*x*_3_) have a piezoelectric sensitivity, which is closely related to the longitudinal piezoelectric coefficient *d*_33_, ranging from 4.7 to 6.4 pC N^−1^.^[^ ^69^ ^]^ Miao et al. investigated the effects of different parameters on the piezoelectric properties of cellulose nanocrystals (CNC).^[^ ^70^ ^]^ They demonstrated that the response depends on the surface chemistry of CNC and on the ionic strength of the solution in which CNC are dispersed. CNC films containing sodium sulfate groups showed a very low piezoresponse, in the order of sub‐pC N^−1^ while films containing hydrogen sulfate groups had a higher *d*_33_ (5.3 pC N^−1^). The authors were able to significantly increase the *d*_33_ to 82.6 pC N^−1^ by casting the CNC films from a suspension 3 mM NaCl. Despite its biocompatibility, cellulose resistance to degradation in numerous organisms, including the human body, poses challenges for its use in biomedical applications, given the lack of specific enzymes (e.g., microbial and fungal) capable of breaking down its strong bonds. Indeed, the numerous intra‐/inter‐molecular hydrogen bonds inside/between polymer chains render the crystalline microfibrils highly resistant to hydrolysis.^[^ ^71^ ^]^

Collagen is the most abundant protein in the human body, comprising ≈30% of the total protein mass. It provides structural integrity and robustness to various tissues, encompassing vital components such as skin, bones, muscles, tendons, and ligaments. Collagen‐rich structures, including cartilage, ligaments, hair, and skin, exhibit a fiber‐like structure with intriguing piezoelectric properties.^[^ ^72^ ^]^ Although the mechanism underlying collagen piezoelectricity has not been fully elucidated, it appears to primarily stem from the inherent molecular charge distribution and polarity within the molecule itself. Residues within collagen molecules feature dipoles that undergo realignment along the molecule's elongated axis when subjected to mechanical stress. This realignment alters the magnitude of the dipole moment, consequently generating piezoelectricity. The piezoelectric coefficient *d*_14_ of collagen ranges from 0.2 to 2 pC N^−1^, a value that can be enhanced through various means, such as adjusting the pH from acidic to neutral. Denning et al. reported a coefficient of *d*_14_ = 12 pm V^−1^ in fibrillary rat tail collagen, indicating the highest piezoelectric response observed among collagen samples.^[^ ^73^ ^]^ Denning et al. also studied the difference piezoelectricity of collagen I and collagen II fibrils.^[^ ^74^ ^]^ Type I collagen is the key component in skin, tendons, and ligaments and it makes up over 90% of the organic material in bones, while type II is mainly found in cartilage. The authors showed that the *d*_15_ of collagen type II was roughly 28–32% of that of collagen type I, speculating that this different piezoelectricity could be related to the different sources of the polymer, to the different polypeptide composition, and to a higher density of covalent crosslinks in type I, which enhance its mechanical stability and reduce its deformability. Techniques aimed at increasing collagen piezoelectric properties include self‐assembling collagen bundles and their cross‐linking through 1‐ethyl‐3‐[3‐dimethylaminopropyl]carbodiimide hydrochloride (EDC)‐*N*‐hydroxysuccinimide (NHS), genipin, and tissue transglutaminase. Bera et al. investigated collagen‐mimicked tripeptide structures, achieving a *d*_33_ coefficient of 4.8 pm V^−1^ and a noteworthy *d*_35_ coefficient (27 pm V^−1^) thanks to the incorporation of hydroxyl groups.^[^ ^75^ ^]^

Chitin is a polysaccharide composed of β‐(1 → 4) linked N‐acetylglucosamine monomers. Chitin is found in the shells of arthropods, cell walls of fungi, and wings of butterflies. The chitin found in crab shells and butterfly wings was found to show piezoelectricity. It may exist in three forms in nature, such as α, β, and γ. In the early 1970s, Fukada et al. discovered the shear piezoelectricity in α‐chitin.^[^ ^76^ ^]^ Hoque et al. utilized biowaste from crab shells to extract chitin nanofibers. The chitin nanofibers used to fabricate a thin film piezoelectric nanogenerator showed a *d*_33_ equal to 9.49 pC N^−1^.^[^ ^77^ ^]^

Chitosan is a natural polysaccharide, which can be extracted from chitin deacetylation. This linear polysaccharide is composed of β‐1,4‐d‐glucosamine. Chitosan exhibits an orthorhombic structure with a nonsymmetric space group of P212121, which endows it with piezoelectric property.^[^ ^78^ ^]^ Chitosan has garnered significant attention in research areas such as biodegradable sensors and energy harvesting systems. The maximum *d*_33_ coefficient reported for chitosan was 18.4 pC N^−1^.^[^ ^79^ ^]^ Amran et al. studied both the *d*_31_ and *d*_33_ of chitosan films, obtained by drying solutions of chitosan in different acids.^[^ ^80^ ^]^ In all cases, the *d*_31_ was found to be higher than the *d*_33_ due to low thickness of the films, enhancing the voltage output for *d*_31_, since in this conformation the piezoresponse was measured perpendicularly to the applied force. Among the different acids, formic acid led to the highest *d*_31_ (10 pC N^−1^) and *d*_33_ (1.1 pC N^−1^). In contrast, films formed from lactic and acetic acid solutions led to the lowest *d*_31_, probably due to a decreased noncentrosymmetry in chitosan in these conditions.

Silk is a natural polymer produced by certain insects and spider species, composed of two different proteins: sericin and fibroin. Silk fibers are composed of fibroin microfibrils arranged into filaments and enveloped by sericin.^[^ ^81^ ^]^ Silk has inherent piezoelectric properties attributed to its intricate α‐helix and β‐sheet substructures.^[^ ^82^ ^]^ Comprising a blend of crystalline and amorphous phases, silk material piezoelectricity is notably influenced by factors such as the degree of crystalline orientation, β‐sheet crystallinity, and an increased presence of silk II.^[^ ^83^ ^]^ Upon subjecting silk films to a draw ratio of 2.7, a shear piezoelectric constant *d*_14_ of ≈1.5 pC N^−1^ was observed. Strategies to enhance silk film piezoelectric characteristics include solution drying, draw, and zone drawing, which improve β‐sheet content and crystallinity.^[^ ^1^ ^]^ In fact, processing parameters have an impact on the piezoelectric properties of silk. Yucel et al. compared two drawing techniques to obtain uniaxially oriented piezoelectric silk films: zone drawing and water immersion drawing.^[^ ^83^ ^]^ Zone drawing at a ratio of 2.7 led to an over twofold increase in the *d*_14_ with respect to untreated films, related to an increase in β‐sheets content and crystal orientation. In contrast, water immersion drawing was less efficient in orienting silk crystals, resulting in a lower piezoelectric response with respect to films obtained by zone drawing. The authors also tried to improve only the β‐sheets content by performing methanol treatment, without aligning the crystals, but this did not result in a piezoelectricity improvement, suggesting that alignment is a key factor in the piezoelectricity of silk.

The filamentous bacteriophage M13 is a single‐stranded DNA virus belonging to the family *Inoviridae*. It has a long, filamentous shape, with a diameter of about 6–7 nm and a length of ≈880 nm. Each phage is enveloped by 2700 copies of a predominant coat protein known as pVIII, alongside five instances of minor coat proteins (pIII and pIX) positioned at both ends. The pVIII proteins exhibit an α‐helical configuration with a dipole moment oriented from the amino‐ to the carboxy‐terminal direction, thereby providing coverage to the phage with a combination of fivefold rotational and twofold screw symmetries. Since the structured alignment of the M13 protein coat lacks inversion symmetry, M13 has inherent piezoelectric properties. Lee et al. measured the piezoelectricity of a film composed of M13 phages, with a thickness > 100 nm, and found a value of *d*_33_ equal to 7.8 pm V^−1^.^[^ ^84^ ^]^ While exhibiting encouraging piezoelectric properties, the complete piezoelectric response of M13 phage films is still not fully understood. A more thorough investigation is needed to completely clarify the phage piezoelectric traits, thereby uncovering its potential applications. Bacteriophages infect bacterial cells; thus, they are considered safe to be used in humans even if recent studies highlighted that they could interact with the host immune system, inducing cellular immune responses.^[^ ^85^ ^]^ This poses some challenges in their direct use in biomedical applications.

#### 2.1.2 Techniques to Improve Piezoelectricity

**Figure 2:**
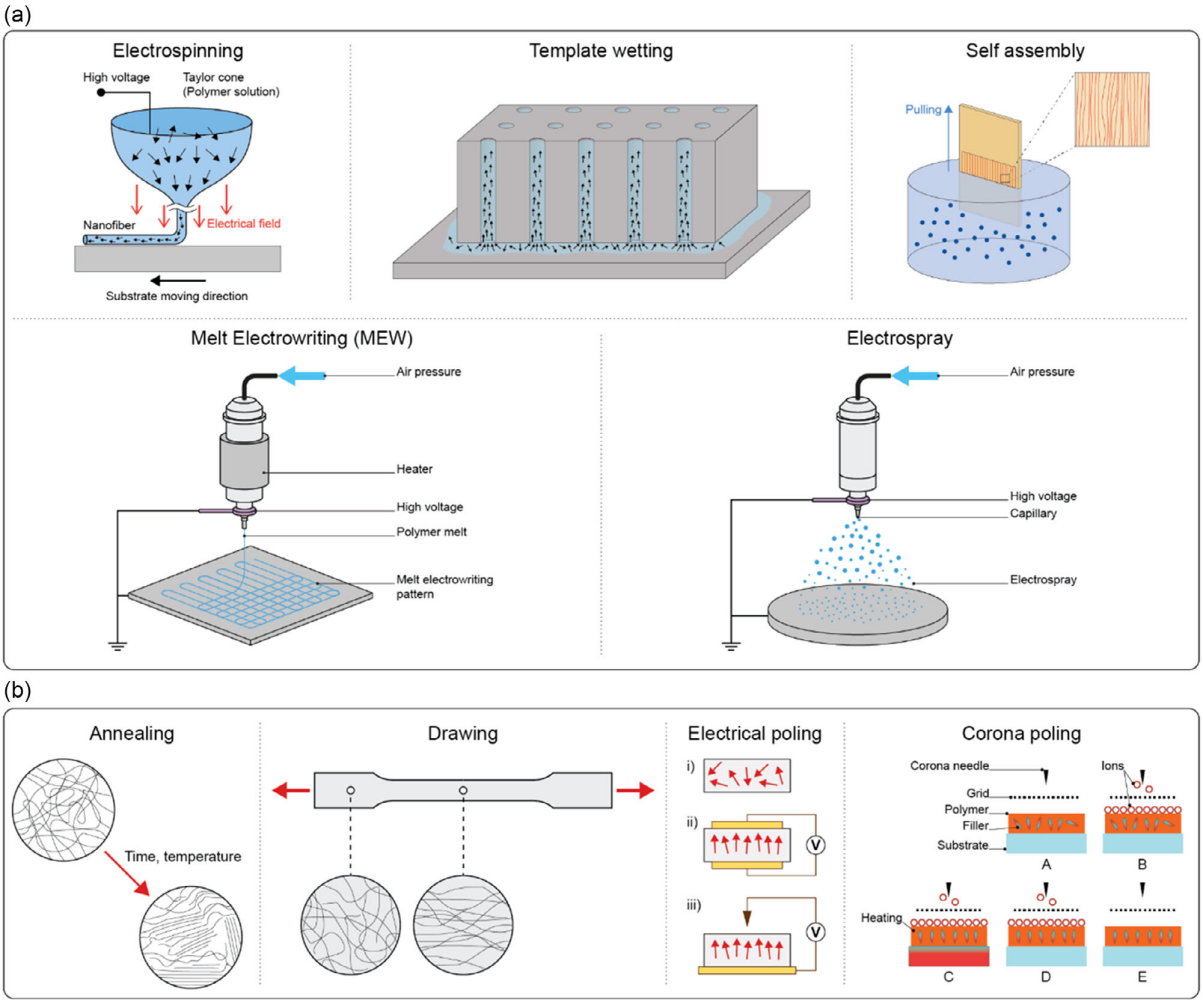
a) Depiction of some techniques used to improve the piezoelectric activity of materials (e.g., electrospinning, template wetting, self‐assembly, melt electrowriting, and electrospray), that tend to favor the alignment of the dipoles in the material, thus maximizing piezoelectricity. b) Polymer crystallization can be controlled in terms of crystal organization as a result of different procedures as: annealing, by adapting time and temperature; drawing a polymer sample, which produces a significantly higher degree of orientation; electrical poling, which allows the alignment of dipole moments in (i) un‐poled ferroelectric material, through the application of an electric field, (ii,iii) with surface electrodes; corona poling of a ferroelectric material, which does not require electrodes attached to the material.

Fabrication techniques for improving piezoelectricity in organic piezoelectric micro and nanomaterials typically involve processes like electrospinning, template wetting, self‐assembly, melt electrowriting (MEW), and electrospray (**Figure** **2**a). Electrospinning is a technique used to fabricate micro‐ and nanofibers by applying a high voltage to a polymer solution or melt.^[^ ^86^ ^]^ The electric field causes the material to be drawn into fine fibers, which are collected on a substrate. These fibers exhibit enhanced piezoelectric properties due to their high aspect ratio and alignment of molecular dipoles during the process. The electrospinning processing and solution parameters can be modulated to obtain the desired morphology and piezoelectric properties. For instance, Tai et al. demonstrated that PLLA concentration played a major role in determining the final fiber diameter, with larger diameters obtained at lower PLLA concentrations; this reflected on the piezoelectric properties of the fibers, since as‐spun fibers with lower diameter displayed larger *d*_33_.^[^ ^87^ ^]^ Gade et al. explored the impact of the applied voltage on the β‐phase content of PVDF electrospun fibers.^[^ ^88^ ^]^ Unexpectedly, the content of β‐phase decreased with increasing applied voltages; the authors concluded that at high applied voltages, the stretching effect provided by the electric field is compensated by the increased jet instability due to the high number of potential ions, leading to a decrease in the electroactive β‐phase. Nevertheless, the effect of the applied voltage on PVDF fiber diameter and β‐phase content is still unclear.^[^ ^89^ ^]^ Other authors, in fact, reported a completely opposite behavior, with an increased β‐phase content at higher applied voltages,^[^ ^90^ ^]^ while others could not observe any dependence at all.^[^ ^91^ ^]^ These differences suggest that the relationship between applied voltage and piezoelectricity is not straightforward, as it also depends on the specific system. Consequently, generalizing a common trend is challenging, and each case must be carefully analyzed under its specific operating conditions. Template wetting involves filling the pores of a template (like anodic aluminum oxide or silicon molds) with a polymer solution.^[^ ^92^ ^]^ After the solvent evaporates, the template is removed, leaving behind micro‐ and nanostructures such as rods, tubes, or wires of the piezoelectric material. This method allows for precise control over the size and shape of the materials, which can enhance their piezoelectric performance.^[^ ^93^ ^]^ Self‐assembly refers to the spontaneous organization of organic molecules into ordered structures, driven by noncovalent interactions such as hydrogen bonding, van der Waals forces, and *π*–*π* interactions. In the context of piezoelectric nanomaterials, self‐assembly can lead to the formation of highly ordered nanostructures, such as micelles, nanorods, or ultra‐thin films, which can exhibit significant piezoelectric properties due to their organized molecular arrangement. Another technique that can be used to fabricate devices based on biodegradable piezoelectric materials together with the self‐assembly approach is the layer‐by‐layer (LbL) deposition. Nanocomposite films can be prepared by LbL to alternately deposit different materials in thin layers, enabling complementary attractive interactions driven by electrostatic forces, hydrogen bonding or covalent bonding.^[^ ^94^ ^]^ The deposition technique depends on the material and on the desired final properties. For instance, a conductive layer can be deposited by sputtering or spin coating, while polymeric films are usually deposited by spin and dip coating or casting. Lee et al. prepared a peptide‐based piezoelectric energy harvester through a meniscus‐driven self‐assembly of aligned FF nanotubes.^[^ ^95^ ^]^ The authors were able to form unidirectionally aligned FF nanotubes by dip‐coating various substrates in a solution of FF in hexafluoroisopropanol (HFIP) and subsequently pulling them at controlled speed. The spontaneous unipolarization of FF nanotubes was attributed both to the interaction with the charged substrates and to the meniscus‐driven self‐assembly. A similar approach was used to fabricate a bio‐piezoelectric nanogenerator based on FF nanotubes.^[^ ^96^ ^]^ FF nanotubes were prepared by dip coating a silicon substrate in a HFIP/FF solution and pulling at a fixed speed. A thin layer of biodegradable polymer (poly lactic*‐co*‐glycolic acid, polycaprolactone, polyvinylalcohol, PLA or PHBV) was then spin coated on the FF nanotubes, and the composite polymer/FF nanotubes membrane was then separated from the silicon substrate and finally a thick Mg electrode was deposited on both sides by an electron beam evaporator. The presence of a polymer matrix, in particular PLA, imparted a high mechanical stress on the FF nanotubes, enhancing the piezoelectric performance of the nanogenerator.

Other techniques that can be used to process piezoelectric polymers include MEW and electrospray. Even though they are not used to improve the piezoelectricity of polymers, they can be employed to process the material to obtain well‐aligned crystal and/or induce dipole during fabrication, which is, in turn, related to piezoelectric properties. MEW is a high‐resolution manufacturing technique based on the electrohydrodynamic processing of polymers used to produce fibers in the micro‐ and nanometer range.^[^ ^97^ ^]^ In MEW, a polymer is first heated up to its melting point into a syringe and then an electric field is applied between the nozzle and the grounded collector plate to draw the polymer as a continuous filament. The gold standard polymer used in MEW is polycaprolactone (PCL); however, other polymers can be also processed with this technique. Florczak et al. were the first to process PVDF with MEW, working at 170 °C and at voltage of 2.7 kV.^[^ ^98^ ^]^ PVDF processed with MEW was found to have an increased β‐phase content up to 79% with respect to the unprocessed polymer (49%), resulting in higher piezoelectric response (*d*_33_ up to 23.7 pm V^−1^). The same group also processed PVDF‐TrFE with MEW, using a similar set‐up.^[^ ^97^ ^]^ Also in this case the content of β‐phase was found to be higher compared to the unprocessed material.

Electrospray is usually used to obtain micro‐ and nanoparticles starting from a solution of the polymer dissolved in a solvent with a certain degree of conductivity.^[^ ^99^ ^]^ The polymer solution is fed through a nozzle or capillary and subjected to a high‐voltage electric field, causing the formation of fine charged droplets. As the solvent evaporates, the polymer forms charged particles. Despite the potentiality of this technique to process piezoelectric biodegradable polymers, to the best of our knowledge it has never been applied to this purpose. Correia et al. produced PVDF microparticles by electrospraying and investigated the influence of the formulation parameters (e.g., polymer concentration, electric field, flow rate) on the size of the obtained microparticles.^[^ ^100^ ^]^ The authors found that the polymer concentration was the most critical parameter for obtaining microparticles, while all the other parameters had minimal impact on the final output. PVDF microparticles had a β‐phase content between 63% and 74%, and a crystallinity between 45% and 55%, regardless of the processing parameters.

However, the piezoelectric response of any material depends on several factors, including the degree of crystallinity, its specific crystalline structure, its morphology, defects, and impurities. Therefore, different techniques can be used to tailor these features to improve the performance of piezoelectric materials. Thermal annealing, drawing, and electrical and corona poling are primarily used for this purpose (Figure 2b).

Thermal annealing is a standard process used to improve the crystallinity of a material and optimize its mechanical, electrical, and thermal properties. The material is heated up to a specific temperature (the annealing temperature), which is usually set above the recrystallization temperature, and then held at that temperature for a defined time. During the heating phase, the material can rearrange its internal structure more efficiently, reducing or even eliminating defects in its crystalline structure. After this phase, the material is cooled down to induce its recrystallization, resulting in a higher crystallinity degree. Thermal annealing, however, does not necessarily improve or act on the anisotropy necessary to guarantee piezoelectricity. In some cases, amorphous structures are key for allowing a piezoelectric response. For example, in odd‐numbered nylon, the piezoelectricity manifests when it displays a disordered mesophase crystal structure, essential for dipole rotation.^[^ ^101^ ^]^ This phase is obtained upon fast quenching of melted nylon. When nylon is annealed, its crystal structure becomes more ordered, thus inhibiting dipole switching, and partially canceling its piezoelectric behavior. In some other cases, annealing can also cause chemical changes, such as the evaporation of constituent elements (i.e., Na, K, Bi), which ultimately lead to the degradation of the piezoelectric properties.^[^ ^102^ ^]^

A technique frequently used to improve the alignment of the dipoles is mechanical stretching (drawing), during which the material, usually a polymer, is subjected to tensile forces to elongate it along the direction of the applied stress. This causes changes in the material's crystalline structure, in the orientation of the polymer chains, and its morphology. The piezoelectric properties of several biodegradable polymers have been enhanced by exploiting this technique. For example, silks and collagens display piezoelectricity because of dipole re‐orientation of their constituent amino acids.^[^ ^103^ ^]^ Kaplan et al. showed that by mechanical drawing silk fibers, the content of ß sheets (rich in electric dipoles) is promoted.^[^ ^83^ ^]^ Indeed, spider silk fibers display enhanced piezoelectricity owing to the increased crystallinity attained by a strain‐hardening process. Authors have also shown that mechanical stretching of a PLLA film up to stretch ratios of 5–6 induced an increase in the *d*_14_ coefficient.^[^ ^104^ ^]^

Another common technique to align the dipole moments in piezoelectric materials is called electrical poling, in which the material is subjected to high electric fields, at high temperatures (close to its Curie point).^[^ ^36^ ^]^ This method is applied to materials that are also ferroelectric, meaning that they possess a spontaneous electrical polarization that can be reversed by the application of an electric field. Electrical poling can be performed in two modalities. In the former, the two opposing surfaces of a piezoelectric material are directly attached to two electrodes. Subsequently, a direct current voltage is incrementally increased until the electric field across the sample aligns with specified saturation polarization values, typically ranging from 5 to 1000 kV cm^−1^. This voltage is sustained for a predetermined duration. At the end, the sample is gradually cooled to room temperature under a constant electric field, and the high‐voltage source is deactivated.^[^ ^105^ ^]^ Recently, Yang et al. demonstrated that by confining the growth of β‐glycine and by in situ poling it during crystallization, films with aligned domains and exceptional piezoelectric performance for this class of biomaterials can be attained.^[^ ^93^ ^]^ In the latter (an indirect method), corona poling is performed. An electrode is put into direct contact with the bottom surface of a piezoelectric material. Above the sample, a pin‐point conduction pointer (the corona needle) operates at high voltages (8–20 kV), inducing the ionization of the gas molecules (either air or inert gas) near the needle. Another metallic grid just below the needle operates at a lower voltage (0.2–3 kV), directing the ionized particles toward the surface of the piezoelectric material.^[^ ^105^ ^]^ Also, corona poling is performed at high temperatures to facilitate the rearrangements of the internal dipoles.

Processing piezoelectric materials with a porous structure can also lead to an increase in their piezoelectric response. For example, Kim et al. showed that nanoporous arrays of PVDF exhibited up to six times higher piezopotential and piezocurrent than those of bulk counterparts.^[^ ^106^ ^]^ It is worth mentioning that an increased porosity can also be detrimental to piezoelectricity. The pore organization can also affect the piezoelectric performance, usually with disordered pores leading to decreased piezoelectricity.^[^ ^107^ ^]^ It is also possible to improve the performance of piezoelectric materials by topological design. Recently, Song and co‐workers have shown that stress redistribution within films can lead to a significant increase in piezoelectricity in kirigami structures of PVDF.^[^ ^108^ ^]^ The authors showed that the stress redistribution can be attained by two different approaches: 1) using laser‐induced thermal topological depolarization; and 2) by rearranging the stress into a specific direction so that the stress is maximized on the surface of the piezoelectric film.

### 2.2 Characterization Techniques

#### 2.2.1 Size, Stability, and Morphology

Characterization techniques are illustrated here to define the piezoelectric nanomaterials’ size, morphology, and stability in physiological fluids, as well as their piezoelectric properties.

Scanning electron microscopy (SEM) and transmission electron microscopy (TEM) are extensively employed to examine the size and morphology of solid micro‐ and nanomaterials or those dispersed in a liquid, following dehydration. SEM is a versatile imaging technique to capture high‐resolution images and detailed surface information from various samples.^[^ ^109^ ^]^ It produces images at higher resolution compared to optical microscopy, with a resolution typically of a few nanometers, employing accelerating voltages that range from 5 to 30 keV. Usually, metallization of samples with a thin layer of gold or platinum is needed to reduce charging effects. TEM can manage an accelerating voltage of up to 300 keV, corresponding to a resolution of tens of nanometers.^[^ ^110^ ^]^ It is a powerful tool for surface texture analysis, allowing for the visualization of atomic structures and lattice arrangements at the nanoscale, thus providing insights into the arrangement of atoms and defects. Also, diffraction studies can be performed to give details on the surface crystallographic structure, identify crystalline phases, determine grain orientations, and analyze defects like dislocations and grain boundaries.

SEM and TEM can also be coupled with energy‐dispersive X‐ray diffraction (EDX), which can be applied to extract qualitative and quantitative information about the elemental composition of the sample. Despite their utility in visualizing materials and distinguishing surface details, these techniques are constrained by a few limitations, including the necessity to dehydrate and metalize the material under investigation. Such processes may alter the surface of the material under visualization. Above all, these methods do not provide information about the three‐dimensionality of nanomaterials or their behavior in a physiological environment.

Atomic force microscopy (AFM) is a nonoptical imaging technique that allows for high‐resolution measurements of various properties of a sample surface in different environments.^[^ ^111^ ^]^ It is particularly handy for the 3D characterization of nanoparticles with sub‐nanometer resolution. To operate AFM, a probe with a sharp tip is scanned over a sample surface using a piezoelectric scanner. As the tip engages the surface, the cantilever bends due to forces between the tip and sample, and this deflection can be utilized for surface topography visualization. This technique can measure the 3D topography (size and shape) and the mechanical properties of nanomaterials, including their elastic moduli.^[^ ^112^ ^]^ This technique is often combined with piezoresponse force microscopy (PFM, described in the next section) to couple topography and piezoelectricity.^[^ ^74^ ^]^ Unlike SEM and TEM, AFM can also be employed in liquid, thus providing information about the material behavior in a liquid environment. Despite these advantages, the use of AFM is limited as the data acquired from the sample are altered by the choice of the tip size, shape and material, which determine different tip‐sample interactions.

While AFM can measure the thickness of nano‐ and microscale materials, its range is limited to heights below 10 μm. Therefore, it is important to consider other techniques that can measure thickness across a broader range, such as standard profilometry, optical profilometry, and spectroscopic ellipsometry.^[^ ^113^ ^]^

Standard profilometry measures the step height between a material and its substrate using a stylus. This method is versatile and straightforward; however, the stylus may damage very soft materials. Optical profilometry, in contrast, is a nondestructive technique that uses interference patterns of light reflected from the material surface and substrate to measure thickness. The thickness is calculated based on the path length differences that produce constructive or destructive interference. While this technique is well‐suited for thin films, its resolution is limited for ultra‐thin films (thickness lower than 100–200 nm), and it relies on the material's refractive index.

Spectroscopic ellipsometry, in contrast, measures changes in the polarization of light (amplitude and phase) reflected from a surface to determine thickness and optical properties. Like optical profilometry, it is nondestructive. However, spectroscopic ellipsometry offers a higher resolution for ultra‐thin films, and a more detailed material characterization, particularly for determining refractive index, optical constants, or the properties of multilayered structures.

Dynamic light scattering (DLS) is a broadly used technique that measures the size and stability of particles in colloidal suspensions. When particles are suspended in a liquid solution, they undergo Brownian motion that causes light to scatter them off. The particles cause localized changes in the refractive index, which produces intensity variations and helps determine their size.^[^ ^114^ ^]^ As a result of DLS, the hydrodynamic size of micro‐ and nanomaterials dispersed in a solution is a measure of their size, while the polydispersity indicates how well they are dispersed (lower values than 0.5 indicate good dispersion^[^ ^115^ ^]^). While DLS is effective in measuring particle size in monomodal samples (e.g., spherical shape), it might not be accurate for anisotropic samples (e.g., nanorods) with a significant difference in the ratio between the diameters of the particles. In such cases, conventional DLS measurements might not be able to measure particle mixtures precisely. In contrast, the DLS technique is often used with electrophoretic light scattering that provide information about the colloidal solution's stability by analyzing the zeta potential, which is related to the surface charge of particles in a colloidal solution. High zeta potential values (positive or negative, absolute value higher than 30 mV) indicate strong electrostatic repulsion between particles. This repulsion helps to prevent particle aggregation, leading to a stable colloidal solution.

Another technique suitable for measuring the size of nanomaterials is the Brunauer–Emmett–Teller (BET).^[^ ^116^ ^]^ BET is a technique used to calculate the specific surface area of materials, particularly porous materials. The method is based on the physical adsorption of gas molecules onto the material's surface. The specific surface area measured by BET can give clues about the shape of micro and nanomaterials. For instance, higher surface area values often indicate more complex or irregular shapes, while lower values might suggest smoother or more spherical shapes.

#### 2.2.2 Piezoelectricity

Various techniques have been employed to investigate piezoelectricity in biomaterials. Laser interferometry has been utilized to evaluate the piezoelectric properties of macroscopic samples by considering the voltage‐induced surface displacement generated by the converse piezoelectric effect. This technique can also furnish data regarding the polarization–electric field (P–E) hysteresis loops, enabling the determination of polarization charge density under the application of DC triangular waveforms.

Second harmonic generation (SHG) imaging is a nonlinear optical procedure wherein two photons of identical frequency interact with a nonlinear medium, generating a photon with twice the frequency (half the wavelength) of the incident photons. This technique evaluates nonlinear optical properties resulting from light diffraction, which is exclusively observed in polar crystals and materials with noncentrosymmetric crystal structures.^[^ ^117^ ^]^ For example, SHG microscopy is widely used in biomedical imaging to visualize collagen fibers in tissues due to its SHG‐active properties.^[^ ^118^ ^]^

As research has shifted from the analysis of bulk materials to thin films, nanostructures, and 2D materials, obtaining information at smaller scales presents challenges and the use of different techniques. Optical‐based methods are limited to the macro‐ and microscopic scale because of the diffraction limit,^[^ ^119^ ^]^ whereas the preparation of electrodes over nanostructures is often unfeasible. Furthermore, natural piezoelectric materials may exhibit weak piezoelectric and ferroelectric properties, which are difficult to identify using macroscopic techniques.

Scanning probe microscopy (SPM) techniques have emerged as one of the most promising approaches to evaluate the piezoelectric and ferroelectric properties at small scales. Among the different SPM methods currently adopted, PFM is the most well‐known technique for elucidating the piezoelectric and ferroelectric properties of micro and nanomaterials.

In PFM, an alternating voltage is applied with a specific frequency to a conductive tip to induce a surface displacement to the material under analysis. The result is an electromechanical response due to the converse piezoelectric effect. From this measurement, the amplitude and phase signals can be deduced, based on the magnitude and polarization direction of the piezoresponse. The estimation of the effective piezoelectric coefficient is based on the linear slope of the plot of amplitude versus Vac amplitude, as piezoelectricity exhibits a linear correlation between mechanical strain and electric field.^[^ ^120^ ^]^ Traditionally, the frequency is selected below the initial contact resonance frequency, which is primarily influenced by the mechanical properties of the cantilever and the tip‐sample contact stiffness.^[^ ^121^ ^]^

PFM hysteresis loop measurements are also commonly employed to examine ferroelectric characteristics. The local electric field‐polarization (E–P) hysteresis can be measured by applying a probing alternate voltage while the direct current voltage is either on‐field or off‐field. It is worth mentioning that the PFM signal is typically obtained in the off‐field state to minimize the electrostatic contribution. This hysteresis loop measurement can be further extended to explore spatially varying local switching behaviors, such as switching spectroscopy‐PFM.^[^ ^122^ ^]^ As a specific operational mode of PFM, the so‐called general mode PFM, allows the acquisition of the cantilever deflection over a full frequency range.^[^ ^123^ ^]^ Multidimensional data can be obtained by simultaneously recording cantilever responses, such as out‐of‐plane and in‐plane deflection, to ascertain the diverse piezoelectric coefficients of the material under analysis. This approach is frequently coupled with nanoindentation modules, which can provide precise control over the force exerted on the material during the stimulation, which is crucial when analyzing biological and soft biomaterials. The peak force tapping allows the probe to intermittently contact the sample to measure instantaneous forces as low as 10^−12^ N. This modality enables the imaging and analysis of synthetic and biological piezoelectric polymers such as cellulose nanowires,^[^ ^124^ ^]^ nylon,^[^ ^125^ ^]^ PLLA,^[^ ^126^ ^]^ and collagen.^[^ ^127^ ^]^

Another PFM approach is based on the utilization of the direct piezoelectric effect.^[^ ^128^ ^]^ Although mechanical stimulation would be more immediate in understanding the piezoelectricity of materials, several drawbacks are still limiting this approach, such as the need for sophisticated electronics to acquire small and reliable electrical signals.

The PFM can provide significant insights into the piezoelectricity at the micro‐ and nanoscale, even if there are drawbacks to approaching similar quantitative values to those measured by macroscopic techniques.^[^ ^129^ ^]^ The occurrence of electrostatic effects has the potential to impact the signal reliability, thereby altering the estimation of the piezoelectric coefficient. In this case, a preliminary analysis through Kelvin probe microscopy can be performed to quantify the surface potential of the material before the PFM analysis. In the alternative, strategies to minimize the electrostatic force include increasing the spring constant of the SPM cantilever,^[^ ^130^ ^]^ conducting measurements at higher AC frequencies,^[^ ^131^ ^]^ and compensating the surface potential by applying an external direct current voltage.^[^ ^132^ ^]^ The use of a stiff tip may partially solve the issue of electrostatic phenomena. Still, such an approach might not be helpful in analyzing organic piezoelectric biomaterials, as many of them are less rigid and resistant than ceramics or synthetic polymers. Another potential source of measurement misinterpretation is caused by the intrinsic dependence on the tip properties, such as stiffness and resonance frequency. Other drawbacks of the PFM analysis include the nonuniform electrical field distribution underneath the SPM tip, as well as instrumental background noise.^[^ ^133^, ^134^ ^]^

PFM is a valuable tool for studying the piezoelectric properties of micro‐ and nanomaterials, but it is still far from being considered an element of industrial test‐bench equipment for standard evaluation, especially for organic materials.

Alternatively, the piezoelectric behavior of a material can be indirectly estimated using other material characterization techniques that are more related to the chemical structure of the polymers.^[^ ^101^ ^]^ Although differential scanning calorimetry (DSC) is not commonly employed to determine the piezoelectricity of polymers directly, it has the potential to provide valuable information about the piezoelectric behavior of polymers indirectly through the analysis of phase transitions and structural changes.^[^ ^87^ ^]^ Polymer phase transitions, such as the glass transition, crystallization, and melting transition, are often associated with changes in molecular ordering and alignment, which can influence the piezoelectric properties of the material. The degree of crystallinity can be quantified using DSC. Crystalline regions within polymers are known to exhibit piezoelectric behavior since the arranged molecular structure aids the generation of electric dipoles in response to mechanical stress. Additionally, DSC can provide insights into the molecular orientation of polymers, which is crucial for understanding their piezoelectric properties. DSC is often used to quantify the crystallinity of thermoplastic materials, such as PLLA.

X‐ray diffraction (XRD) can also provide information about the crystallographic structure and molecular arrangement of polymers, which are fundamental factors influencing their piezoelectric behavior.^[^ ^2^, ^79^ ^]^ By analyzing the diffraction patterns, XRD can determine the presence and nature of crystalline phases within polymer samples, which is responsible for the piezoelectric behavior. XRD can also detect structural changes induced by mechanical deformation or external stimuli, providing information on the improvement of the piezoelectric properties induced on the biomaterial. Indeed, polar group alignment along specific crystallographic directions can enhance the piezoelectric properties of polymers by promoting a preferential orientation of electric dipoles in response to mechanical stress. XRD is usually used to analyze the presence of different phases in organic materials such as glycine.^[^ ^93^ ^]^

A further helpful method for determining the existence of specific crystal phases is the Fourier‐transform infrared (FTIR) spectroscopy. FTIR spectroscopy can identify the functional groups in polymers based on their characteristic absorption bands in the infrared spectrum.^[^ ^135^ ^]^ Specific functional groups, such as polar groups (e.g., —OH, —NH2, —COOH), or hydrogen bonding interactions within or between polymer chains are known to contribute to the piezoelectric properties of polymers by facilitating the generation of electric dipoles in response to mechanical stress. This method can also allow to check for polymer chain alignment within the sample by employing a polarizer. FTIR is often used to discriminate the different phases of PVDF. Still, it has also been explored for the analysis of the secondary structures of proteins at the micrometer scale (e.g., formation of β‐sheets in silk). Similarly to FTIR, Raman spectroscopy is another powerful tool for investigating the piezoelectric properties of materials. It may provide detailed information about the vibrational modes of a material, which can be correlated with its piezoelectric behavior. Raman spectroscopy can be used to identify different crystalline phases of a material.

Although DSC, XRD, FTIR, and Raman spectroscopy do not directly measure piezoelectric properties, they can be used with other techniques, such as polarized light microscopy, mechanical testing, and piezoelectric measurements, to provide a comprehensive understanding of the piezoelectric behavior of polymers.

A summary of the techniques discussed is reported in **Table** **2**.

**Table 2:** Summary of the techniques used to characterize piezoelectric biomaterials from the morphological, piezoelectric, and chemical perspective.

Technique	Sample type and preparation	Instrument parameters	Sample size	Outputs
Electron microscopy	Solid and dried sample coated, covered by a thin layer of gold/platinum for nonconductive samples (not needed for environmental Electron microscopy). For TEM of organic nanomaterials, a staining with uranyl acetate might be necessary; for larger samples it is often required the use of the ultramicrotome	SEM: acceleration voltage (typical range: 5–30 keV), probe current, spot size, working distance, detector type (secondary electron detector or backscattered electron detector), scanning speed and vacuum level (low or high) TEM: acceleration voltage (typical range: 50–300 keV), condenser lens system (size and intensity of the electron beam), objective lens focus and aperture, camera length, detector type, vacuum level, stigmation modulation	SEM: lateral dimensions of few μm up to 1–2 cm in size; Thickness: lower than few mm TEM: lateral dimension: lower than 3 mm per dimension; Thickness: less than 100 nm	Size and morphology, elemental composition, atomic structures, lattice arrangements, arrangement of atoms and defects, surface crystallographic structure and crystalline phases, grain orientations
Atomic force microscopy (AFM)	Dried or liquid samples in the form of thin films, or micro‐ and nano‐sized materials. No special preparation needed	Tip shape and radius, tip coating, cantilever stiffness, cantilever resonant frequency, scanning speed, tip‐sample interaction mode (contact, noncontact or tapping), force or amplitude setpoint, scan area	Area: 0.25–0.5 μm up to 100–200 μm; Thickness: sub‐nm to hundreds of μm, with steps lower than 10 μm in the topography	3D topography (size and shape), mechanical properties of nanomaterials, (e.g., stiffness), and other surface properties (e.g., adhesive, electrical and chemical properties)
Dynamic and electrophoretic light scattering	Suspension of solid particles	Laser wavelength, scattering angle, detector sensitivity, temperature, measurement duration, cuvette material, and path length, applied voltage	Volume: 1–3 mL Particle size: 0.5 nm to 10 μm Concentration: typical range from 10^−5^ (w/v) up to 10% (w/v), dependent on material optical properties (e.g., absorbance or refractive index)	Hydrodynamic size, stability (polydispersity index)
BET	Dried samples in the form of thin films, or micro‐ and nano‐sized materials	Adsorption gas (e.g., nitrogen, krypton), relative pressure (typical P/P_0_ range: 0.05–0.35), degassing temperature and time	Sample size: 10–500 mg, depending on the instrument and the surface area	Specific surface area (m^2^ g^−1^), pore volume and pore size distribution, adsorption‐desorption isotherms
Piezoresponce force microscopy (PFM)	Dried samples in the form of thin films, or micro‐ and nano‐sized materials, over a conductive substrate	Tip shape and radius, tip coating, cantilever stiffness, cantilever resonant frequency, drive amplitude and phase, scanning speed, tip‐sample interaction (applied force), scan area	Area: 0.25–0.5 μm up to 100–200 μm Thickness: sub‐nm to hundreds of μm, with steps lower than 10 μm in the sample topography	3D topography (size and shape), and piezoelectricity (amplitude and phase signals)
Differential scanning calorimetry (DSC)	Fine powder, film, or small piece of solid materials	Temperature range, heating/coaling rate, sample pan type, purge gas and flow rate, instrument sensitivity, and resolution	Sample size: from 1 to 10 mg	Thermal properties: phase transitions, melting points, crystallization, and specific heat capacity
X‐ray diffraction analysis (XRD)	Dehydrated solid samples, powders, thin films, or small bulk pieces, deposited onto a specific sample holder with zero background	X‐ray source, X‐ray tube voltage (10–60 kV), and current (5–100 mA), goniometer settings (e.g., scan range, step size, and scan speed), detector type, slits, temperature	Area: the area typically scanned is around 1–5 mm^2^ Thickness: few nm up to 10–50 μm	Crystallographic structure and molecular arrangement of polymers, elemental composition, surface details
Fourier‐transform Infrared Spectroscopy (FTIR)	Dried and liquid samples, powders, thin films, or coatings	Spectral range (typically 100–4000 cm^−1^) and resolution, detector type, source type, interferometer configuration (e.g., Michelson interferometer), path length, number of scans, beam splitter material	Area/volume: up to 1 cm^2^ with thickness above 200–300 nm (thin film). A few mg for solid samples, or a few μL liquid solutions	Vibrational modes of molecules, chemical composition, and molecular structure, functional groups in polymers or hydrogen bonding interactions
Raman spectroscopy	Dried and liquid sample, powders, thin films, or coatings, and microparticles	Excitation wavelength (commonly 532, 633, or 785 nm), laser power, spectral range (typically 100–4000 cm^−1^) and resolution, collection mode (backscattering vs transmission), numerical aperture and magnification of objective lens, integration time, scanning speed and accumulation, temperature control, filter and optical components, environment control, detector type, signal averaging, background subtraction	Area: small sample areas (micron‐scale) can be analyzed; no strict size requirement for bulk samples	Vibrational modes of molecules, chemical composition and molecular structure, functional groups in polymers and crystalline information

#### 2.2.3 Biodegradability

The concept of “degradation” refers to an irreversible alteration in the overall characteristics, structure, and form of the polymer, often induced by the chemical breakdown of its molecular chains.^[^ ^136^ ^]^ More generally, degradation is driven by biotic means (biodegradability) or abiotic means (hydrolysis, photolysis, or oxidization). Biodegradation is a multifaceted and naturally occurring phenomenon influenced by the biological activity of living organisms.^[^ ^137^ ^]^ By definition, a biodegradable material is a substance that biological processes can naturally decompose into simpler and nontoxic compounds. For the applications addressed in this review, the state‐of‐the‐art report refers to the biodegradation of piezoelectric micro‐ and nanomaterials in the human body. Thus, in this context, biodegradability strictly refers to the material's ability to be processed within the body and divided into nontoxic subunits over the average human lifespan. More specifically, ISO‐10993‐9‐2009 defines “biodegradation” as the “decomposition of a material due to the biological environment”.^[^ ^138^ ^]^

Conducting in vitro degradation tests is crucial for estimating degradation rates and providing a basis for in vivo experimentation. The primary forms of degradation observed in vivo are hydrolytic and enzymatic degradation. It is of pivotal importance to first define the operating conditions of the piezoelectric material, such as temperature, pH, and presence of specific enzymes or microorganisms, to mimic the environment in which the material is expected to degrade. The material is then placed in a controlled environment and regularly monitored to check critical parameters such as weight loss, changes in physical or chemical properties (e.g., piezoelectricity) and the production of degradation byproducts to determine the rate and extent of degradation. In general, biodegradation proceeds by two complementary processes: bulk degradation and surface degradation erosion. Bulk degradation involves the cleavage of molecular bonds, leading to the breakdown of the polymer into smaller molecular fragments and a gradual degeneration of the molecular weight and mechanical properties of the material. In contrast, surface degradation entails the loss of mass from the surface of the polymeric matrix, primarily through the dissolution and diffusion of the smaller fragments, often via hydrolysis, oxidation, or microbial degradations.^[^ ^139^, ^140^ ^]^

The standardization of biodegradation in vitro studies is challenging due to the vast array of approaches utilized, resulting in only a limited number of standards addressing general testing requirements. For instance, ISO 10993 ‐ Part 9^[^ ^138^ ^]^ and Part 13^[^ ^141^ ^]^ offer a standardized platform for the characterization and quantification of potential degradation products in polymeric medical devices via in vitro degradation studies. In particular, Part 13 outlines general specifications for sample preparation, suitable test conditions, and procedures for measuring initial mass under varied environmental conditions. Additionally, it suggests employing diverse characterization techniques to assess different properties of polymeric biomaterials such as solution viscometry to evaluate the average molecular mass and branching, rheology to determine the mechanical properties, chromatographic methods (e.g., gas and/or high‐performance liquid chromatography) for residual monomers, gel permeation chromatography (GPC)/size exclusion chromatography (SEC) for molecular mass averages and changes in molecular mass distribution, mass spectrometry for identification of byproducts, spectroscopic methods (e.g., ultraviolet spectroscopy, infrared spectroscopy, nuclear magnetic resonance, mass spectroscopy) for identity, composition, distributions, and thermal analysis (e.g., DSC) for glass transition, melting range or softening point. Besides ISO 10993, ISO 13781 provides guidelines specifically tailored to study the in vitro degradation of implants for surgery based on poly(lactide) homopolymers, copolymers, and blends.^[^ ^142^ ^]^ Also, in this case, the tests are based on monitoring the loss of the sample mass, variations in the molar mass through inherent viscosity and gel permeation/size exclusion chromatography, and variations in mechanical properties.

The selection of an appropriate liquid solution is highly pivotal to effective degradation testing. Most degradation investigations documented in the literature,^[^ ^143^, ^144^, ^145^ ^]^, as also outlined in ISO 13781,^[^ ^142^ ^]^ are conducted using phosphate‐buffered saline (PBS) solutions. However, it is worth mentioning that employing PBS may primarily induce degradation via hydrolysis. Consequently, alternative and more reliable degradation solutions that mimic the composition of the targeted environment are also utilized, including ionic solutions, enzyme buffers, and simulated body fluid solutions, to broaden the understanding of degradation mechanisms and the degradation rate of the material under analysis.

Quantifying the mass loss of the biomaterial is a primary crucial aspect to assess degradation. This procedure involves determining the initial mass of the sample and comparing it with measured mass values obtained during degradation. This test involves periodic material rinsing to avoid stationary steady state, usually performed with distilled or deionized water to eliminate impurities, loose particles, or residual buffer solution. Then, the sample is vacuum‐dried to remove any residual moisture and attain a constant weight.^[^ ^146^ ^]^ While this assessment is commonly feasible for macrostructures, in the case of micro‐ and nanomaterials, specific investigation methods are needed to determine the mass variation over time. Quartz crystal microbalance or thermogravimetric analysis can be used to measure mass changes up to the nanogram level by monitoring the frequency changes of a quartz crystal resonator or by measuring the mass change of a sample as it is heated, providing information on thermal stability and composition, respectively. However, other techniques may provide indirect mass variation by analyzing the volume through a topographical image at the nanoscale (e.g., AFM), or by measuring the size distribution of small particles in suspension, from which mass can be inferred if the density is known (e.g., DLS). Alternatively, SEM provides detailed images of materials, and EDX can be used to determine the elemental composition. Combined, they can give an estimate of mass based on the material's volume and density.

During the degradation of polymer‐based materials, the molecular weight of the polymer may decrease over time due to the breakdown of the molecular bonds. This variation can be monitored by viscosity measurements and GPC/SEC, as reported in ISO13781.^[^ ^142^ ^]^ Viscosity is linked to molecular weight, as polymers with higher molecular weight tend to increase the viscosity of the solvent in which they are dissolved. The average molecular weight can be determined from the intrinsic viscosity using the Mark–Houwink equation^[^ ^147^ ^]^

(2) $$\left[\eta \right]=K\left(M_{v}\right)\alpha$$

where [η] represents the intrinsic viscosity, M_ν_ denotes the viscosimetric molecular weight, and *K* and *α* are constants dependent on the polymer, solvent, and temperature. Alternatively, GPC/SEC can assess degraded molecular weight distributions by discriminating molecules in a sample based on their size, and this measurement may be applied to monitor changes in the molecular weight of polymers. First, the polymer is dissolved in a suitable solvent. Then, it is injected into a column filled with a porous inert material; as the sample passes through the filler, molecules of larger size can only penetrate a short distance into the filler due to their restricted access to deeper regions. Conversely, molecules of smaller size can penetrate deeper into the filler, resulting in their later elution from the chromatographic column. In the end, a refractive index detector can distinguish molecules with different molecular weights; thus, chains of different sizes are segregated into distinct compartments of the filler, allowing for the simultaneous evaluation of various molecular weights based on elution times.

In addition to tracking mass loss (and morphology) and changes in molecular weight, alterations in mechanical properties, surface chemistry, thermal behavior, water absorption, and environmental conditions are also undertaken, depending on the features of the biomaterial and its applications. To this end, various characterization techniques are employed, including DSC for thermal analysis, XRD for assessing crystallinity, X‐ray photoelectron spectroscopy for surface chemistry analysis, and FTIR spectroscopy for identifying functional groups.^[^ ^144^, ^145^ ^]^

Finally, an indirect method to study the degradation of a biomaterial is tracking the production of degradation byproducts. In this case, the analysis should be performed on the solution in which the material is immersed for the test, and the chemistry of the degradation reaction should be known to track specific chemicals with the appropriate technique. For example, proteins and peptides are degraded into smaller units, i.e., amino acids. The presence of free amino acids in the solution indicates degradation. The concentration of these molecules in the solution can be measured over time with several techniques, such as UV–vis or fluorescence spectroscopy (for optically active molecules in the UV–vis range), or by high‐performance liquid chromatography associated with mass spectroscopy.

While the ISO standards mentioned here provide guidelines on how studying degradability in vitro, the target for the optimal degradation time should be evaluated case by case, as it strongly depends on the application. More specifically, in tissue engineering and regenerative medicine, the degradation time should not be shorter than the healing or regeneration process.^[^ ^148^ ^]^ For instance, a slow degradation rate is usually required for bone regeneration since it may take about 6–12 weeks for significant bone healing.^[^ ^149^ ^]^ In contrast, faster degradation kinetics might be desirable where the complete resorption of the biomaterial is needed in a short time (e.g., absorbable surgical sutures made by PLA fibers degrade within 60 days from the implantation^[^ ^150^ ^]^).

While piezoelectric ceramics do not present any specific degradability characteristics, piezoelectric biomaterials can undergo different degradation processes depending on their chemical structure.

PVDF and its copolymer PVDF‐TrFE are not considered biodegradable in natural environments or under physiological conditions.^[^ ^98^ ^]^ These materials are synthetic fluoropolymers, known for their chemical stability, thermal resistance, and durability, and their resistance to degradation is due to the strong carbon–fluorine bonds in their structure, which are among the strongest bonds in organic chemistry.

The biodegradability of both PLA and PLLA has been extensively demonstrated in the literature. Degradation of PLA and PLLA within the body primarily occurs through hydrolysis of their ester‐bond backbone, forming lactic acid monomers or oligomers that may be further decomposed as final metabolites in water and carbon dioxide.^[^ ^151^ ^]^ The kinetics of degradation are usually slow (up to 2 years), and the rate depends on environmental factors, such as pH and temperature of the tissue, or on the molecular features of the polymer, such as its molecular weight and its chirality (PLLA degrades more slowly than PLA). In a study on the long‐term degradation of implants composed of high molecular weight PLLA in rats, Bos et al. observed a decrease in polymer mass and molecular weight initiated 3 months after implantation, attributed to pure hydrolysis.^[^ ^152^ ^]^

The biodegradation kinetics of PHB in vivo depend on many factors, including the composition and metabolic activity of microbial consortia, environmental factors like pH, temperature, and oxygen concentration, as well as intrinsic properties of the polymer, such as its molecular weight and degree of crystallinity.^[^ ^153^ ^]^ PHB exhibits a slower degradation rate within physiological settings than PLLA. Meischel et al. demonstrated that rats’ composite PHB bone implants do not degrade entirely within 36 weeks in vivo.^[^ ^154^ ^]^

PMLG can undergo enzymatic or hydrolytic degradation, where enzymes or water molecules cleave the peptide bonds within the polymer backbone. PMLG degradation primarily occurs through enzymatic hydrolysis, where specific enzymes break down the polymer into smaller units through the cleavage of ester bonds within the polymer chain. Esterases, particularly those produced by microorganisms, play a significant role in this degradation mechanism.^[^ ^155^ ^]^ The hydrolysis of the ester bonds in PMLG leads to the release of L‐glutamic acid derivatives.

Piezoelectric materials based on amino acids and peptides can be biodegraded into fundamental molecules when exposed to a biological environment without inducing toxic effects in the host system. For instance, a study by Hosseini et al. demonstrated that a piezoelectric film composed of glycine and chitosan was dissolved entirely in PBS within 48 h of immersion.^[^ ^156^ ^]^ However, the degradation kinetics of these materials are heavily influenced by the water solubility inherent to the peptide or amino acid constituents.

Chitin and chitin‐based materials like chitosan are renowned for their notable resistance to degradation through nonenzymatic pathways such as acidic and oxidative degradation; however, they exhibit susceptibility to biodegradation in vivo through hydrolysis by lysozyme, an enzyme available in mammals,^[^ ^157^ ^]^ and through the action of chitinases, which are glycosyl hydrolases responsible for cleaving the β‐(1‐4)‐linkages present in the N‐acetyl‐D‐glucosamine units constituting chitin polymers.^[^ ^158^ ^]^ Chitosan can degrade within days to weeks when exposed to chitosanases and other specific enzymes. The rate is influenced by the degree of deacetylation and the molecular weight of the chitosan. Chitin is generally more resistant to degradation than chitosan due to its higher degree of acetylation. Specific chitinases can break it down, but this process can take weeks to months, depending on enzyme concentration and environmental conditions.^[^ ^159^ ^]^

Collagen‐ and silk‐derived biomaterials are susceptible to enzymatic decomposition, requiring catalysis for effective breakdown into aminoacids or other smaller fragments, that can metabolized by transamination or deamination, within a physiological environment.^[^ ^160^ ^]^ Enzymes such as carboxylase, actinase, and chymotrypsin have demonstrated efficacy as catalysts for degrading silk polymers.^[^ ^161^ ^]^ In vitro investigations have elucidated that while the amorphous component of silk can undergo rapid degradation within the initial days of exposure to enzymatic solutions, the highly organized crystalline phase necessitates a more prolonged period, typically around 15 days, for decomposition to occur. The degradation mechanism in vivo involves a longer timeframe, often months to years, albeit without triggering an immunogenic response. Studies by Wang et al. highlighted differential degradation behaviors of silk scaffolds implanted in Lewis rats and nude mice, indicating that modifications in processing methods and crystallinity levels can influence biodegradation rates.^[^ ^162^ ^]^ Collagen has faster degradation kinetics in vivo when compared to silk‐based materials. Alberti et al. conducted a study investigating the biodegradation of highly oriented collagen fibrils in Sprague–Dawley rats, revealing that vulcanization with glutaraldehyde significantly improved stability (up to 3 weeks), while nonvulcanized collagen scaffolds exhibited rapid decomposition within 21 days.^[^ ^163^ ^]^

About peptides, glycine is a fundamental nutrient involved in neurological function, metabolic regulation, and antioxidant processes. It is readily catabolized and primarily absorbed in the small intestine, where the glycine cleavage system facilitates its breakdown, producing ammonia and CO_2_.^[^ ^164^ ^]^ In contrast, diphenylalanine may be degraded by peptidases or proteases as primary degradation pathway, that induces hydrolysis to phenylalanine. A secondary route may be represented by nonenzymatic hydrolysis or oxidation, leading to aromatic intermediates or other byproducts.^[^ ^36^ ^]^

The kinetics of M13 phage degradation strongly depends on the environment in which they are used. In vitro studies showed that M13 phages were biodegraded entirely within 45 min from their exposure to artificial gastric juice; in contrast, within the same timeframe, the extent of M13 phage degradation was less pronounced in other fluids (e.g., 88% in saliva, 66% in urine, and 44% in blood) (**Table** **3**).^[^ ^165^ ^]^

**Table 3:** Comparison of biodegradable piezoelectric organic micro‐ and nanomaterials in terms of type of material synthesis approach, size and shape, biodegradation time, and biodegradation mechanism in physiological conditions.

Material	Fabrication approach	Shape and size	Biodegradation medium	Biodegradation time	Biodegradation mechanism/byproducts and immune reaction	References
Poly‐L‐lactic acid (PLLA)	Solvent‐casting	Thin film, thickness: 100 nm (size: 18 × 18 mm^2^)	Degradation test at 37 °C in 50 mM Tris‐HCl buffer (pH: 8.5) with 0.2 mg mL^−1^ of proteinase K	Amorphous regions completely degraded after 120 min, and only the crystalline regions remained intact	Enzymatic degradation	[239]
	Dip coating	Thin film, 50–150 μm (Eco‐PLLA 520)	Degradation test at 37 °C in Sorensen's phosphate buffer (pH 7.4)	Molecular weight almost halved over time, while the weight loss over 23 weeks was lower 10%	Hydrolytic degradation	[240]
	Compression molding	Thin film, thickness: ≈35 μm	Degradation test at 37 °C in PBS for 14 days, then switched to 74 °C. Implantation in the backs of mice (C57BL/6J model)	No significant degradation of the PLLA sensors after 14 days. The sensor completely degraded at 74 °C after 56 days In vivo, the sensor did not degrade	Hydrolytic degradation Mild immune reaction without significant presence of inflammation, multinucleated giant cells, and fibrous capsules	[241]
	Electrospinning	Electrospun mat, thickness: 19–28 μm (size: 5 × 5 mm^2^)	Implantation on a craniotomy defect in the mouse's skull (C57BL/6J model)	Degradation after 4 weeks of implantation	Hydrolytic degradation of the ester‐bond backbone The device elicits minimal fibrosis and immuneresponse	[225]
Polyhydroxybutyrate (PHB)	Electrospinning	Electrospun mat, thickness: 250 μm	Degradation test at 37 °C in PBS	PHB showed a 6 % mass loss after 4 weeks	Hydrolytic degradation	[242]
	Electrospinning	Electrospun mat, size 50 × 50 mm^2^ Fiber diameter: 196 ± 65 nm	Degradation test at 37 °C in simulated body fluid with and without lysozyme (concentration not reported)	Weight loss is about 1.8% after 30 days. In the presence of lysozyme, weight loss increases to 14.4% in 30 days	Hydrolytic or enzymatic degradation	[243]
	Dip coating	Thin film, thickness: ≈100 μm (size: 10 × 10 mm^2^)	Degradation test at 37 and 70 °C in Sorensen's buffer (0.1 m, pH 7.4), with and without pancreatin (concentration not reported) Implantation in a defect (6 mm in diameter) in the abdomen of male Wistar rats	Molecular weight decreased by 10, 25, and 45% after 3, 6, and 12 months. Accelerated degradation induced by pancreatin (molecular weight decrease of 30% after 3 months). Absence of changes in film mass over 52 weeks In vivo, the material had a molecular weight of about 38% of the initial value after 26 weeks	Hydrolytic degradation. Enzymatic catalysis is unclear as the degradation behavior consists of molecular weight loss but constant mass	[244]
γ‐Glycine	Casting with solvent evaporation	Thin film, thickness: ≈70 μm (with Mb electrodes and PLA encapsulation)	Degradation test at 37 °C in PBS Implantation in the back of the mice (C57 model)	Dissolution of PLA‐encapsulated γ‐glycine/PVA film within 2 weeks In vivo, the material was gradually absorbed within 10 days.	Degradation mechanism not reported No evidence of tissue damage, immune response, or recognizable material byproduct	[186]
	Casting with solvent evaporation	Thin film, thickness: 30 μm (with Mb electrodes)	Implantation under the skin in the dorsal region of rat (Sprague Dawley rat model)	The device completely disappeared after 1 day	Degradation mechanism not reported. Absence of any significant reference to immunorespoinse	[245]
β‐Glycine	Drop casting and solvent evaporation	Thin film, thickness: 38 μm	Degradation test at room temperature in PBS	The glycine/chitosan film dissolved after 2 days	Not reported	[156]
Poly‐γ‐methyl‐L‐glutamate (PMLG)	Dry spinning	Spun fibers, thickness not reported	Degradation test at 37 °C in PBS with the pronase E (concentration not reported)	Dissolution within 15 h, depending on the concentration of pronase E	Hydrolytic and enzymatic degradation	[246]
Poly‐γ‐benzyl‐L‐glutamate (PBLG)	Not reported	Thin film, thickness not reported	Degradation test at 37 °C in PBS with a protease (type IV, activity: 1.0 unit mg^−1^).	Degradation lower than 3% after 40 days	Hydrolytic degradation	[247]
Diphenylalanine (FF)	Self‐assembly by dip coating	Self‐assembled monolayer of fibers, thickness not reported	Degradation test at 37 °C in PBS	Dissolution after 10 min	Hydrolytic degradation	[248]
	Self‐assembly and spin coating	FF microrods (diameter: 2–15 μm; length: ≈21 μm) in a PLA matrix (thickness: ≈51 μm)	Degradation test at 60 °C in PBS, 0.6 M NaOH, or 0.6 M HCl solutions	Complete dissolution in all media after 25 days. The degradation rate is in the order of alkaline solution > acidic solution > phosphate‐buffered saline	Hydrolytic degradation	[236]
Collagen	Not reported	Film thickness: ≈40 μm (noncrosslinked fibrillar bovine collagen I)	Subcutaneous implantation in adult male Lewis rats	Decrement in the film thickness of ≈75% in 21 days	Enzymatic degradation	[249]
	Not reported	Film thickness: 400 μm (porcine skin‐derived collagen: DHT and DHT/EDC cross‐linked membrane)	Degradation test at 37 °C in a 0.25% porcine trypsin solution. Subcutaneous implantation in albino male Wistar rats	Decrease in the thickness of the DHT and DHT/EDC membranes in 4 weeks after implantation (≈halved their thickness). In all cases, the thickness was close to 100 μm after 12 weeks	Enzymatic degradation	[250]
	Commercial material	Geislich Bio‐Gide (Geistlich Biomaterials), thickness: 400 μm (25 × 25 mm^2^)	Implanted under the skin of the dorsal part of the cranium in Wistar‐derived rats	Degradation of 60% after 4 weeks, and of 80% after 9 weeks	Enzymatic degradation	[251]
	Commercial material	Geislich Bio‐Gide (Geistlich Biomaterials), thickness: 400 μm	Implantation on intracranial defects of Wistar rats	The membrane thickness halved after 30 days	Enzymatic degradation	[252]
Chitin	Casting	Thin films of chitin with different degrees of deacetylation (from 0% to 100%), thickness: 150 μm	Degradation in PBS with lysozyme (4 mg mL^−1^). Implanted in the subdermal tissue of Wistar rats back	Pure chitin: 20% remaining weight after 30 h. Other deacetylated derivatives: weight remaining from 50% to 90% after 30 h In vivo, chitin and chitin with 69% degree degraded quite rapidly (50% after 2 weeks). while other deacetylated derivatives resisted up to 12 weeks, (weight remaining higher than 50%)	Enzymatic degradation	[157]
	Casting	Thin film, thickness: ≈35 μm, β‐chitin	Degradation at room temperature in 1 UN/10 mL chitinase, in deionized water	Chitin film completely degraded after 8 days. The degradation time decreased to ≈4 days with 5 UN/10 mL of chitinase	Enzymatic degradation The material might generate natural by‐products during biodegradation, such as CO_2_ gas	[253]
Chitosan	Drop casting and solvent evaporation, crosslinked with NaOH	Thin film, thickness: 38 μm	Degradation at room temperature in PBS	Mg electrodes degraded within the first minutes in PBS, while glycine/chitosan completely dissolved after 2 days	Not reported	[156]
	Casting	Thin films, thickness: 150 μm	Degradation in PBS with lysozyme (4 mg mL^−1^) Implanted in the subdermal tissue of Wistar rats back	Pure chitosan lost less than 10% of its mass after 30 h In vivo, chitosan resisted with a negligible mass loss over 12 weeks	Enzymatic degradation	[157]
Keratin	Self‐assembly	Thin film, thickness: 30–40 μm (size: 1 × 1 cm^2^)	Degradation at 37 °C in a solution with trypsin Subcutaneous implantation in the back of a male mouse (Nippon Clare, Jcl:ICR 10 W)	Degradation achieved 40–50% within 2 weeks, then leveled off at 50–60% in the following 18 weeks In vivo, slower degradation kinetics, with a linear tendency that extent up to 60% over 18 weeks	Enzymatic degradation	[254]
Silk	Spinning	Spun fiber, thickness not reported	Degradation at 37 °C in PBS with and without Protease XIV (1.0 mg mL^−1^)	Mass loss higher than 50% after 42 days with protease XIV. In PBS, the fibers did not degrade	Proteolytic degradation (enzymatic degradation)	[255]
	Casting	Thin film, thickness: ≈0.5 mm (size: 3 × 3 cm^2^)	Degradation at 37 °C in 0.05 M sodium phosphate buffer (pH 7.0), with ant without α‐chymotrypsin, collagenase IA, and protease XIV (1.0 U mL^−1^)	Weight rapidly decreased to 70% in 1 day in all cases. After 15 days, the weight of the sheet was 68, 48, 30, and 68% of the initial mass in α‐chymotrypsin, collagenase IA, protease XIV, and phosphate buffer, respectively	Proteolytic degradation (enzymatic degradation) Degradation products from collagenase IA protease XIV reported a molecular weight lower than 2.4 kDa, while those in the buffer without enzymes ranged from 20.0 to 70.0 kDa	[256]
	Forming	Thin film, thickness: 100 nm	Degradation at 37 °C in PBS with protease XIV and α‐chymotrypsin (enzyme concentration: 300 μg mL^−1^)	α‐chymotrypsin did not contribute to significantly degrading the silk crystals over 24 h. Protease XIV formed nanofibrils, and decreased the thickness of silk crystals from 5 to 2 nm over 24 h	Proteolytic degradation (enzymatic degradation) Degradation products from protease XIV contained several low molecular weight fragments (less than 50 kDa) with respect to the α‐chymotrypsin	[257]
Cellulose	Not reported	Thin film, thickness 3 mm	Implanted subcutaneously in female Wistar rats	No evidence of degradation in vivo up to 12 weeks	Nonbiodegradable in physiological conditions	[258]
M13 bacteriophage	Enforced infiltration	M13 phage with a rod shape, diameter: 6.6 nm; length: 880 nm	Degradation in human blood, saliva, urine, artificial gastric juice (AGJ)	Decrease of phages in human blood, saliva, and urine by 44, 88, and 66% after 45 min, respectively. No phage resists after 5 min in AGJ. PBS do not show significant decrease over time	Proteolytic degradation (enzymatic degradation)	[165]

#### 2.2.4 Biodegradability: From a Cellular Perspective

An interesting scenario would be represented by the internalization of piezoelectric nanomaterials within cells and the subsequent intracellular digestion. To guarantee nanomaterial internalization, the size should be <100 nm for modulating the intracellular processes because a smaller size is required for endocytic cell uptake.^[^ ^166^, ^167^ ^]^ In contrast, >100 nm may be preferred if cell uptake is not desired, such as for the stimulation of the external side of the plasma membrane for neural cell activation.^[^ ^12^ ^]^ Also, spherical nanomaterials are often internalized more efficiently than other shapes due to their symmetry, while for rod‐shaped nanomaterials, the internalization may occur at a slower rate and dependently on the aspect ratio. Also, materials with sharp edges may face more resistance in membrane internalization.

The internalization of nanomaterials within cells has demonstrated beneficial effects when triggered by ultrasound (US) waves.^[^ ^19^, ^168^ ^]^ In particular, nanoparticle‐mediated effects induced by US mainly refer to trigger intracellular response in terms of Ca^2+^ and Na^+^ fluxes or the generation of reactive oxygen species (ROS), which can alter intracellular pathways with up or down‐regulation of genes and/or proteins.^[^ ^169^ ^]^ However, the knowledge of their fate requires further research from a biodegradation perspective.

Intracellular digestion of nanomaterials involves the cellular uptake, processing, and degradation of these materials within the cell. This process can be pretty complex, involving various cellular mechanisms to manage and break down foreign particles. Nanomaterials enter the cell through endocytosis, a process by which cells entrap external elements that can be differentiated into phagocytosis, pinocytosis, and receptor‐mediated endocytosis.^[^ ^170^ ^]^ Nanomaterials can also be delivered directly to the cell membrane by biochemical or physical means. In this situation, they can freely target subcellular organelles and intracellular structures, as endosomes do not mediate internalization. However, endocytosis is the path most approached to target piezoelectric nanomaterials within the cells.

Once the nanomaterial is inside the cells, it remains enclosed in an endosome, a membrane‐bound vesicle, that will fuse with a lysosome, forming a phagolysosome. Lysosomes are organelles filled with acidic hydrolase enzymes capable of breaking down various biomolecules. Indeed, within the lysosome, the nanomaterials are exposed to an acidic environment (pH around 4.5) and various enzymes, such as proteases, lipases, nucleases, and glycosidases.^[^ ^171^ ^]^ These enzymes attempt to degrade the nanomaterials. However, the degradation's extent depends on the nanomaterials’ chemical composition and structure. Piezoelectric nanomaterials, often made from materials like PZT, BTO, or ZnO, may resist complete breakdown due to their robust inorganic nature.^[^ ^166^, ^172^ ^]^ In the specific case of polymeric piezoelectric materials, they can be altered according to their chemistry and sensitivity to enzymatic degradation.

The degradation process generates byproducts that can be thoroughly degraded, resulting in soluble molecules that the cell can utilize, expel, or partially degrade, leading to smaller nanoparticles or ions that the cell needs to manage.^[^ ^173^ ^]^ If the nanomaterials or their degradation byproducts cannot be fully broken down, they may be expelled from the cell through exocytosis. This process involves the vesicle containing the waste materials moving toward the cell membrane and merging with it, releasing the contents into the extracellular space and thus circulating.

The intracellular digestion of nanomaterials is a multistep process involving cellular uptake, endosomal and lysosomal processing, enzymatic degradation, and potential exocytosis. During this period, the nanomaterial must execute its task proficiently for the duration of the protocol under investigation. The presence and digestion of piezoelectric nanomaterials within cells may have various biological impacts, such as inflammatory response, oxidative stress, or cytotoxicity. Thus, understanding these mechanisms is crucial for evaluating piezoelectric nanomaterials’ biocompatibility and potential therapeutic applications.

From this perspective, using biodegradable piezoelectric nanomaterials aims to maximize biocompatibility and biodegradability for safer intracellular digestion and integration into cellular metabolism, reducing cytotoxicity and potentially harmful biological impacts in the human body. Indeed, biodegradable polymers often possess functional groups that can interact with cell surface receptors.^[^ ^174^ ^]^ For instance, polysaccharides can bind to carbohydrate‐recognition receptors, while amino acid‐based polymers can interact with specific protein receptors.

Once internalized in endosomes and lysosomes, different enzymatic degradation modalities can be carried out, such as proteolytic, glycolytic, lipolytic, and nucleolytic. In the case of protein and amino acid‐based polymers, materials are broken down by proteases into smaller peptides and amino acids. Then, these smaller molecules can be further processed or utilized in cellular metabolism. In the case of polysaccharides, materials can be hydrolyzed by glycosidases into monosaccharides (e.g., glucose) and oligosaccharides. These simpler sugars can be used in metabolic pathways like glycolysis or stored as glycogen.^[^ ^173^ ^]^

Indeed, degradation products from biodegradable polymers can often be integrated into cellular metabolism, providing an energy source or building blocks for biosynthesis. In the alternative, undegraded or partially degraded materials that cannot be integrated are packaged into vesicles and expelled from the cell through exocytosis.

## 3 Applications of Biodegradable Piezoelectric Nanomaterials

### 3.1 Applications in Tissue Engineering and Regenerative Medicine

Studies on the use of biodegradable piezoelectric micro‐ and nanomaterials are still in their infancy, probably due to the limitations imposed by their smaller piezoelectric coefficients with respect to inorganic counterparts. Nevertheless, some examples available in the literature show their potential in tissue engineering and regenerative medicine.

In a recent work, Xia et al. used piezoelectric PLLA nanofibers to stimulate the differentiation of neural stem cells into neural cells (**Figure** **3**a).^[^ ^175^ ^]^ The fibrous scaffolds were prepared by electrospinning an 8% w/v PLLA solution, followed by a coating with polydopamine (PDA) for 3 h performed in an ultrasonic cleaning machine. Such a coating enhanced the hydrophilic properties of the material, improving its biological interaction with cells without altering the piezoelectric properties. Interestingly, the PDA‐coated PLLA fibers (diameter: 500–700 nm) exhibited the largest cell spreading area at 430 μm^2^, compared to 200 μm^2^ for the uncoated PLLA. Quantitative analysis revealed that about 70% of the scaffold area on PDA‐coated PLLA was covered by cells after seven days, compared to only 25% on pristine PLLA. The neural stem cell differentiation was significantly enhanced on PDA‐coated PLLA, with higher gene expression levels for NG2, Olig2, GFAP, Tuj1, and NeuN. This phenomenon was explained by speculating that the nanofiber network underwent restructuring due to the formation of focal adhesion between the cells and nanofibers, with consequent deformation of the piezoelectric nanofiber, which, in turn, generated an electrical signal.^[^ ^176^ ^]^ This stimulation modified the local membrane potential, causing the opening of voltage‐dependent channels, allowing calcium ions to enter, and ultimately facilitating gene transcription to regulate stem cell differentiation.^[^ ^177^ ^]^ Using finite element analysis, the authors showed significant deformation on a single PLLA nanofiber due to cell traction, with a maximum stress of 20 kPa and a maximum piezoelectric potential of 10.11 mV.

Figure 3Application examples of organic piezoelectric biomaterials embedded in polymeric matrices for tissue engineering and regenerative medicine. a) Cell expansion and neural differentiation influenced by adhesion conditions, material type (tricalcium phosphate and poly‐L‐lactic acid), and chemical functionalization. The surface‐induced effects vary with the electric potential generated by stress on oriented piezoelectric nanofibers during cell movement, leading to enhanced neural differentiation, particularly in the presence of poly‐L‐lactic acid nanofibers and polydopamine. Reproduced with permission.^[^ ^175^ ^]^ Copyright 2022, Wiley. b) Variation in heat treatment affects the phase transition between electrospinning‐induced amorphous and crystalline α/α' phases in poly‐L‐lactic acid, influencing piezoelectric performance in both transverse and longitudinal directions. This tuning of piezoelectric properties in poly‐L‐lactic nanofibers is crucial for controlling stem cell differentiation, with neurogenesis and osteogenesis being enhanced by orthogonal and shear piezoelectric effects, respectively. Reproduced with permission.^[^ ^87^ ^]^ Copyright 2021, Elsevier. c) Schematic of glycine‐polycaprolactone nanofibers with uniformly distributed glycine particles across the poly caprolactone matrix. The piezoelectric glycine‐poly caprolactone offers a promising platform for glioblastoma therapy and medical implant development. Reproduced with permission.^[^ ^183^ ^]^ Copyright 2023, The American Association for the Advancement of Science. d) The piezoelectric hydrogel contains short piezoelectric nanofibers of poly‐L‐lactic acid within a collagen matrix, designed for injection into knee joints under arthroscopic or X‐ray guidance for osteoarthritis treatment. Ultrasound activation of the hydrogel generates electrical cues mediated by the nanofibers (visible in red within the collagen network). Reproduced with permission.^[^ ^187^ ^]^ Copyright 2023, Nature Publishing Group. e) Scheme of the diphenylalanine‐based nanogenerator fabrication process, alongside images of the diphenylalanine nanotubes. The nanocomposite matrix generates piezoelectricity through mechanical strain from cell migration over the substrate. Reproduced with permission.^[^ ^96^ ^]^ Copyright 2023, Elsevier.

**Figure:**
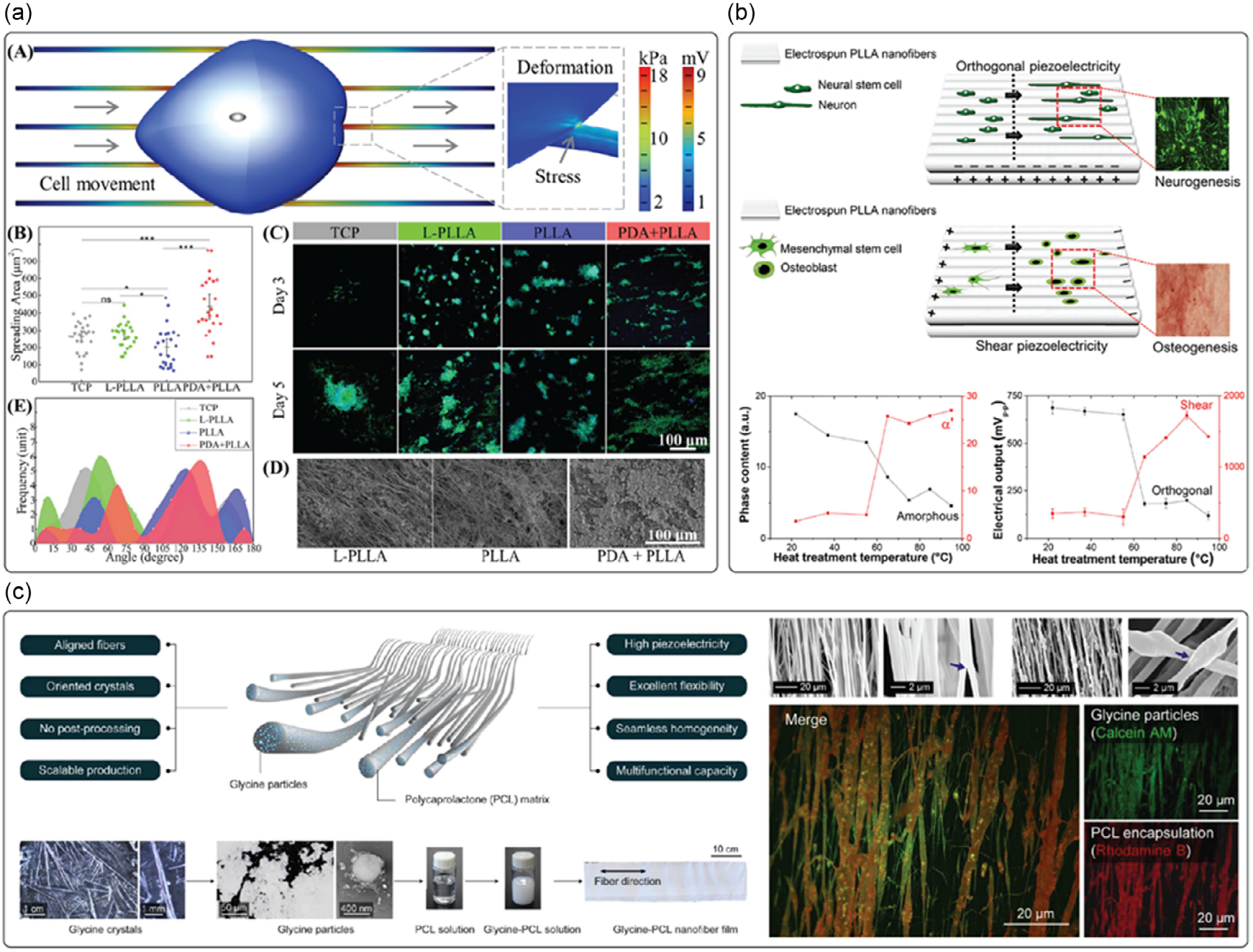

**Figure:**
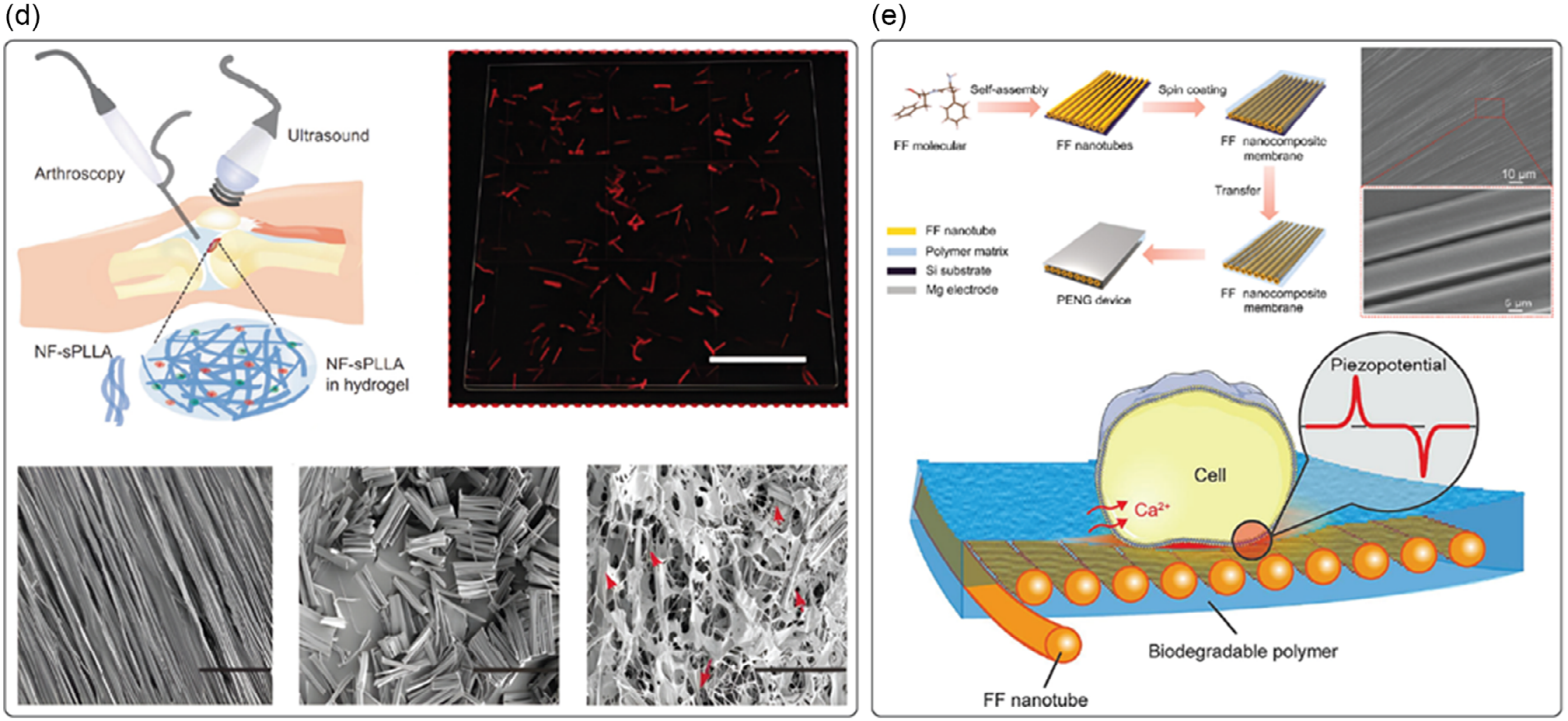

The differentiation of neural stem cells on electrospun PLLA nanofibers was also investigated by Tai et al. (Figure 3b).^[^ ^87^ ^]^ In their work, the authors explored the correlation between the piezoelectric properties of aligned PLLA nanofibers with fiber diameters ranging from 30 to 500 nm and the efficiency of stem cell differentiation in a phenotype‐specific manner. Also in this case, the authors speculated that PLLA‐mediated piezoelectricity was induced by the deformation of the scaffolds induced by cell contractile forces. Several PLLA fibers with different diameters and that had undergone different heat postprocessing procedures were investigated. It was demonstrated that the differentiation efficiency strongly depended on the piezoelectric orientation. As‐spun PLLA nanofibers having a fiber diameter of 150 nm, a thickness of 30 μm, and longitudinal piezoelectricity (*d*_33_ ≈ 5 pm V^−1^) favored neural differentiation of human neural stem cells due to mechanical contraction forces generated by the cell‐matrix interaction through focal adhesion complexes, developing a local piezoelectric potential 0.5–10 mV, sufficient for cell activation. The neural cells cultured on nanofibers demonstrated an upregulation in the expression of neuronal genes, specifically NGN2, MAP2, and ENO2, which correspond to early, intermediate, and mature neuronal markers, respectively. These cells also exhibited a significantly higher levels of β3‐tubulin, a prominent neuronal marker. In contrast, osteogenic differentiation of human mesenchymal stem cells was favored on heat‐treated PLLA nanofibers with preferential shear piezoelectricity; in this case, the piezoelectric effect would be induced by the shear force generated by cell elongation and migration. The findings indicated that calcium deposition matched the increased levels of osteogenic differentiation markers such as RUNX2, ALPL, SPP1, and BGLAP in the heat‐treated electrospun nanofibers. Overall, the author proposed electrospun PLLA nanofibers with regulated piezoelectric properties for developing self‐powered platforms for engineering stem cell into specific phenotypes.

Liu et al. tested a biodegradable piezoelectric scaffold made of PLLA nanofibers, both in vitro and in vivo, to promote chondrogenesis and cartilage regeneration in the treatment of osteoarthritis.^[^ ^178^ ^]^ The PLLA nanofiber‐based scaffold was fabricated with a thickness of about 19 μm. Thanks to its piezoelectric properties, the PLLA scaffold, under force or joint load, produced a manageable piezoelectric charge, leading to increased expression of COL2A1, ACAN, SOX9, higher deposition of glycosaminoglycans and high adsorption of extracellular proteins (i.e., fibronectin) in adipose‐derived stem cells (ADSCs) cultured directly on piezoelectric scaffolds in vitro. In contrast, the scaffold facilitated migration or recruitment of cells, triggering the endogenous TGF‐β pathway through calcium signaling, and ultimately enhancing chondrogenesis and cartilage regeneration in vivo. Rabbits with critical‐sized osteochondral defects treated with the piezoelectric scaffold and under an exercise regimen demonstrated hyaline cartilage regeneration within 1–2 months of exercise. In contrast, rabbits treated with a nonpiezoelectric scaffold but still under the exercise regimen showed unfilled defects and limited healing.

Apart from nanofiber‐based scaffolds, such as those based on PLLA, the incorporation of piezoelectric nanomaterials into polymeric matrices represented an alternative approach to endowing the matrix with piezoelectric properties. Several examples of inorganic piezoelectric nanoparticles embedded in polymeric matrices have been reported so far.^[^ ^179^, ^180^, ^181^, ^182^ ^]^ In many cases, composites were defined as biodegradable even if only the polymeric matrix was the degradable component, thus including inorganic fillers such as BTO, or organic as graphene oxide. Recently, scientists have begun to look at including biodegradable piezoelectric micro and nanomaterials into composite matrices as their performance has increased due to the application of fabrication techniques to improve their piezoelectric coefficients. To date, only a few articles have reported the use of biodegradable piezoelectric biomaterials as fillers of polymeric matrices.

One of the most investigated organic piezoelectric fillers is glycine. There are a few examples in the literature, mainly referred to as β‐glycine and γ‐glycine. In the former case, a biodegradable piezoelectric sensor composed of crystallographically oriented β‐glycine spherulites embedded in an amorphous chitosan polymer was proposed by Hosseini et al. (Figure 3c).^[^ ^156^ ^]^ Briefly, a composite solution of glycine/chitosan at a ratio of 0.8:1 w/w (chitosan: 1.5% w/v) was drop cast onto a Petri dish and dried at room temperature for 24–48 h to obtain films with a thickness of 38 μm. The sensor produced a 190 mV output voltage under 60 kPa pressure with a sensitivity of 2.82 ± 0.2 mV kPa^−1^ (stable signal up to 9000 cycles), and wholly degraded within 48 h in physiological solution (PBS). Chorsi et al. reported a novel strategy to fabricate biodegradable, flexible, and piezoelectric matrices by embedding β‐glycine crystals powder within PCL nanofibers,^[^ ^183^ ^]^ to address the limitations of brittle solvent‐cast films made exclusively from glycine crystals (Figure 3d). The authors investigated the electrospinning process to fabricate soft and degradable composite PCL fibers (diameter: 2 μm) by adding 500 nm‐grounded β‐glycine crystals as filler (concentration ratio: 1:1 w/w), in which the effective piezoelectric constant *d*_33_ measured with a piezometer resulted in the range 18.4–20 pC N^−1^, comparable to the KNN‐based materials (*d*_33_ = ≈20 pC N^−1^).^[^ ^184^ ^]^ The prospective application was for a biodegradable implantable US transducer for opening the blood‐brain barrier in drug delivery applications. Indeed, the glycine‐PCL composite film demonstrated stable piezoelectric performance with a high ultrasound output of 334 kPa under 0.15 voltage root‐mean‐square (*V*_rms_). This application led to a remarkable twofold increase in animal survival time in mice with orthotopic glioblastoma models.

Regarding the γ‐glycine, Yu et al. proposed a γ‐glycine‐polyethylene oxide (PEO) composite (ratio: 3:1 w/w) film with a sandwich structure activable by applying mechanical force for generating electrical signals.^[^ ^185^ ^]^ The γ‐glycine‐PEO films were obtained by drying a drop‐casted composite, with solvent evaporation at 40 °C for 48 h. Such a device showed an out‐of‐plane *d*_33_ of ≈8.2 pC N^−1^ which is close to the theoretical *d*_33_ value of the pristine γ‐glycine crystals (10.4 pC N^−1^),^[^ ^61^ ^]^ and a degradation time of 10 min in PBS.

More recently, a flexible and biodegradable wireless US electrotherapy device based on a composite of γ‐glycine and poly(vinyl alcohol) (PVA) was proposed by Xue et al.^[^ ^186^ ^]^ The authors demonstrated the self‐alignment and orientation of γ‐glycine encapsulated by PVA layers (glycine/PVA ratio: 2:1), which provided enhanced piezoelectric properties, with a *d*_33_ coefficient of 10.4 pC N^−1^. This composite film (thickness: ≈70 μm) generated a US‐induced wireless outputs of about 220 mV mm^−1^, and showed interesting results in reducing wound healing time by ≈40% in in vivo animal models compared to controls.

Vinikoor et al. reported an injectable piezoelectric hydrogel comprising PLLA nanofibers and collagen type I designed for osteoarthritis treatment (Figure 3e).^[^ ^187^ ^]^ The PLLA nanofibers were produced through electrospinning to form aligned nanofiber mats that were annealed to enhance crystallinity, then cut into short fibers (length: ≈25 μm; diameter lower than 1 μm), and mixed with collagen to formulate the injectable hydrogel. The PLLA nanofibers‐generated voltage upon US stimulation. Under accelerated conditions (80 °C), the hydrogels quickly degraded, broke down, and lost their original structures, while at physiological temperature (37 °C) they slightly reduced their volume within 2 months. In vitro investigations revealed that the piezoelectric hydrogel promoted cell migration, triggered TGF‐β1 secretion, and upregulated gene expression associated with chondrogenesis. In animal models, US‐activated piezoelectric hydrogel facilitated the regeneration of new cartilage and subchondral bone, similar to that of healthy cartilage.

Other recent examples of nanocomposite materials made of biodegradable polymer matrices, as poly(lactic*‐co*‐glycolic acid) (PLGA), PVA, poly (3‐hydroxybutyrate*‐co*‐3‐hydroxyvalerate) (PHBV), PCL, and PLA including FF nanotubes has been reported as a novel bioresorbable energy harvester (Figure 3f).^[^ ^96^ ^]^ The FF nanotubes (diameter up to 7 μm) were prepared by dip‐coating a silicon wafer substrate into a FF/co‐solvent solution (ratio 3:1, co‐solvent solution: 1,1,1,3,3,3‐Hexafluoro‐2‐propanol and double‐distilled water) at a pulling speed of 60 μm min^−1^. Nanocomposite membranes were fabricated by coating the polymeric solutions with FF nanotubes on the silicon wafer substrate, and using a spin coater to obtain a final thickness of around 20 μm. FF was featured by a maximum piezoelectric coefficient *d*_15_ of 55.18 pm V^−1^ (10 mg mL^−1^). These platforms showed a high level of degradability, mainly dependent on the chemistry of the polymeric matrix tested: the composite made of PLGA completely degraded in 70 days, while those made with PLA and PHBV lost up to 30% of mass after 120 days. The potential benefits of using such nanocomposite materials for tissue engineering were evaluated by examining the effect on the alignment and proliferation of human fibroblasts over 3 days. In vitro, evidence showed that the piezoelectrical potential generated by cell migration can activate the ion channel to induce the Ca^2+^ transmission rate, in turn promoting cell metabolism and proliferation.

The fundamental mechanism of piezoelectricity, involving the alignment of dipoles within a material, remains consistent across both micro‐ and nanoscales. However, piezoelectric micro‐ and nanomaterials operate at different scales and exhibit unique properties due to their size. Nanomaterials, for instance, possess a significantly higher surface area‐to‐volume ratio, resulting in enhanced surface‐related properties such as increased surface charge distribution, mechanical strength, and flexibility.

In cellular applications, nanofibers are often favored due to their nanoscale interactions with cells, which can influence cellular behavior, including topographical organization and differentiation. Organic materials with notable shear piezoelectric coefficients have been extensively studied, partly due to their compatibility with fabrication techniques like electrospinning (e.g., PLLA) or the formation of anisotropic structures through self‐assembly (e.g., FF). Notably, FF and glycine (β‐ and γ‐polymorphs) when encapsulated have demonstrated the ability to enhance the piezoelectric response of relatively inert but biocompatible polymeric matrices such as PCL or PVA. This encapsulation facilitates interactions between the piezoelectric material and the substrate or polymeric coating, promoting uniaxial polarization and increasing the piezoelectric response.

Conversely, microsized thin films have been primarily explored as transducers due to their significant *d*_33_ coefficients, achieved through the inclusion of piezoelectric micro‐ and nanofillers like glycine and FF. These materials hold promises for applications ranging from biodegradable sensors to electrotherapy.

### 3.2 Applications in Targeted Therapy and Small‐Scale Robotics

Piezoelectric micro‐ and nanomaterials have recently been used in biomedical applications, primarily due to two key features. The first aspect is their ability to become electrically polarized remotely, which can be achieved, for example, by using acoustic waves^[^ ^19^ ^]^ or combining piezoelectric building blocks with magnetostrictive components to form strain‐mediated magnetoelectric composites.^[^ ^188^ ^]^ In the latter case, electrical polarization of the piezoelectric part is triggered by external magnetic fields, which is another suitable approach to activate these materials once implanted in the body remotely. Several authors have successfully demonstrated the enhanced proliferation and differentiation of different cell types using acoustically or magnetically activated piezoelectric (and magnetoelectric materials), including neuronal cells, osteoblasts, adipose cells, muscle cells, and endothelial cells.^[^ ^169^ ^]^ Pané and co‐workers created porous degradable scaffolds consisting of PLLA honey‐comb networks containing magnetoelectric nanoparticles and demonstrated enhanced proliferation of osteoblasts upon magnetoelectric stimulation (**Figure** **4**a).^[^ ^189^ ^]^ Nogués and co‐workers used flexible layers of magnetostrictive iron‐gallium and piezoelectric PVDF on Kapton and confirmed the feasibility of magnetoelectrically differentiating osteoblasts.^[^ ^190^ ^]^ Maturation and mineralization were also confirmed. Das et al. have recently shown that biodegradable PLLA‐based self‐charged piezoelectric scaffold could effectively induce skin regeneration in mouse models. Additionally, it was revealed that these materials displayed antibacterial activity against *S. aureus* and *P. aeruginosa*.^[^ ^191^ ^]^ Piezoelectrically induced differentiation mechanisms are typically triggered by interactions with the calcium channels in the cell membranes. For example, Hoop et al. utilized a piezoelectric polymer PVDF substrate that generates electrical charges on its surface upon acoustic actuation with US to stimulate the differentiation of PC12 cells.^[^ ^192^ ^]^ Performing inhibitor experiments, they demonstrated the role of a cyclic adenosine monophosphate‐dependent pathway in the generation of neurites, indicating that the piezoelectric actuation of PVDF by ultrasonic waves activates calcium channels.^[^ ^12^ ^]^

Figure 4Examples of organic piezoelectric biomaterials in targeted therapy and small‐scale robotics. a) Illustration of the effect of magnetic stimulation on cells cultured on 3D magnetoelectric scaffolds under alternating current magnetic fields. Inorganic nanoparticles dispersed in poly‐L‐lactic acid exhibited ferroelectric and magnetoelectric properties, as confirmed by numerical simulations, which are crucial in triggering cell proliferation into the scaffold. Reproduced with permission.^[^ ^189^ ^]^ Copyright 2019, Elsevier. b) Schematic of piezoelectric hybrid lipid‐polymeric nanoparticles that efficiently encapsulate a nongenotoxic drug (nutlin‐3a) and are functionalized with a peptide (ApoE) to enhance blood‐brain barrier penetration. These nanoparticles induced Ca^2+^ influx in ultrasound‐stimulated cells and promoted cell migration within 24 h. Reproduced with permission.^[^ ^196^ ^]^ Copyright 2022, Elsevier. c) Illustration of hybrid nanoeels based on polyvinyl fluoride and their swimming behavior characterization. Time‐lapse images show movement in response to varying amplitude and frequency of the applied magnetic field, alongside a drug release profile triggered magnetically by the hybrid nanoeels. Reproduced with permission.^[^ ^21^ ^]^ Copyright 2019, Wiley. d) Filler‐modification approach for ultrasound‐activated poly‐L‐lactic acid membranes, designed to optimize PLLA crystallinity and chain orientation for effective cell piezostimulation. An example of this application is the piezostimulation of HaCaT cells, which leads to heat spread and temperature changes before and after stimulation on poly‐L‐lactic composite films. Reproduced with permission.^[^ ^211^ ^]^ Copyright 2023, Wiley.

**Figure:**
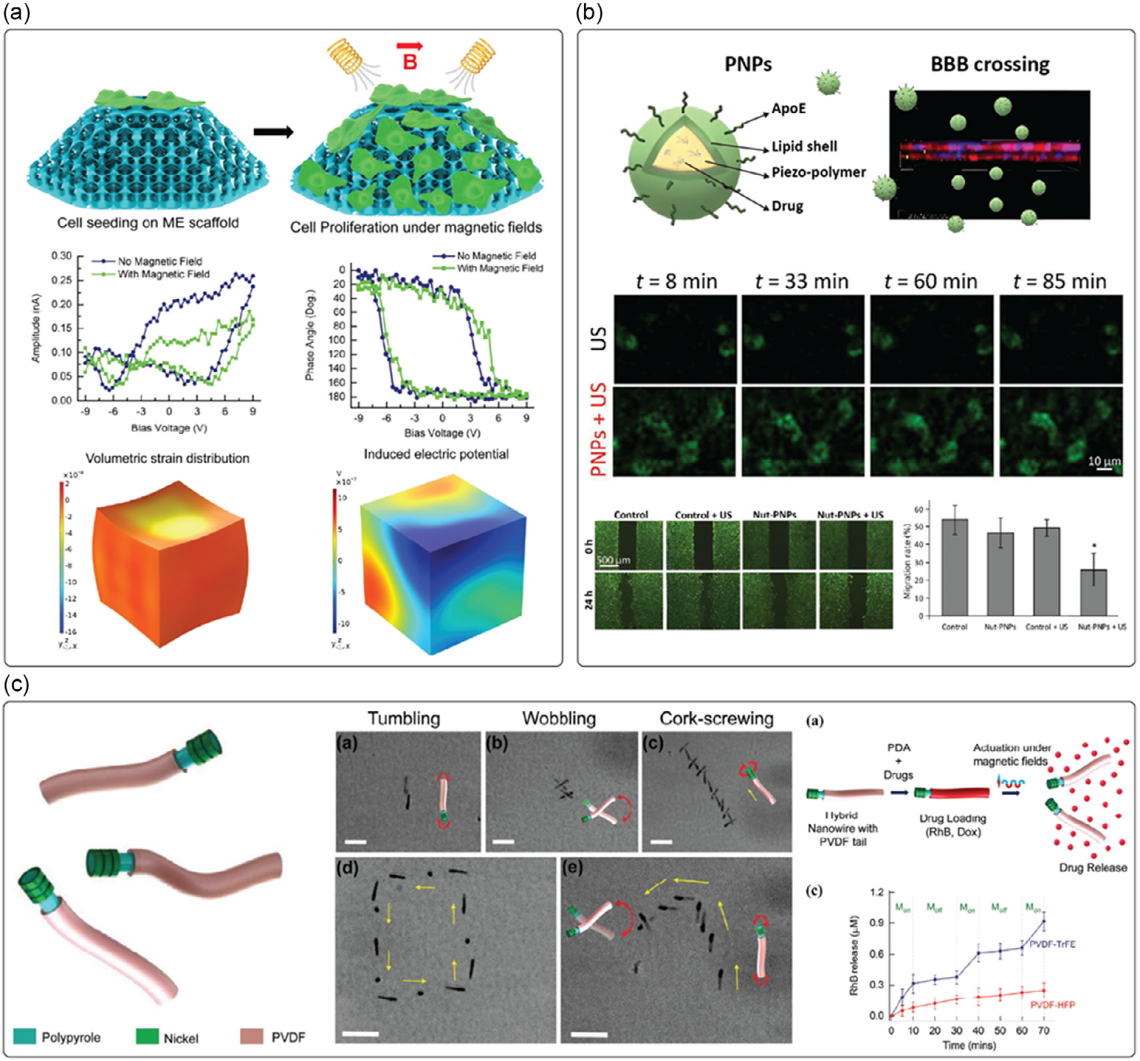

**Figure:**
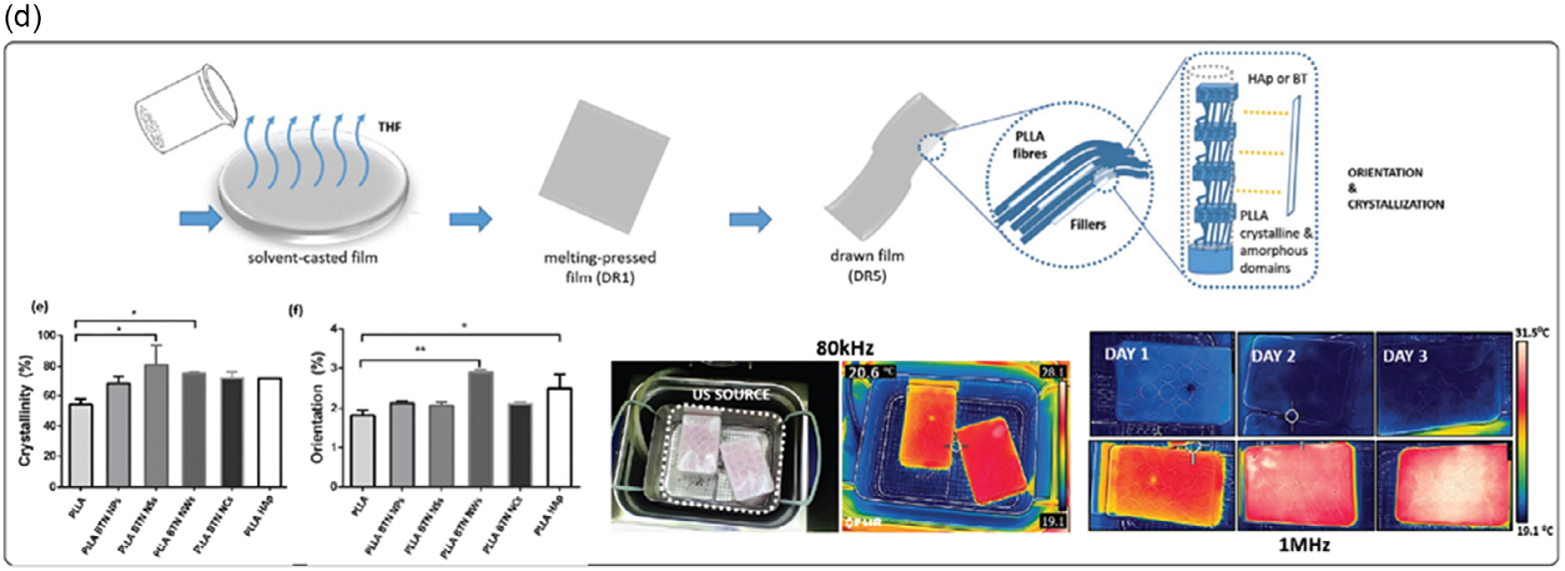

The second key feature of piezoelectric materials in biomedical applications is their capability to induce catalytic processes when their surface is interfaced with water. This phenomenon, known as piezocatalysis, can generate ROS in water, such as OH^•^ and O_2_^–•^– radicals, which can be used for tumor therapies.^[^ ^193^ ^]^ Several researchers have tested the efficacy of piezocatalysis in vitro and in vivo (rodents) in tumor treatment, for example, in breast cancer, skin cancer, or glioblastoma, using nondegradable piezoelectric nanomaterials.^[^ ^194^ ^]^ Recently, Qi and co‐workers utilized BTO nanoparticles as a noninvasive electro‐treatment to inhibit tumor growth in triple‐negative breast cancer, demonstrating that the therapeutic efficiency was positively correlated with the intensity of electric stimulation.^[^ ^195^ ^]^ Additionally, Pucci et al. introduced a method employing US‐responsive drug‐loaded organic piezoelectric nanoparticles – i.e., nutlin‐3a‐loaded ApoE‐functionalized P(VDF‐TrFE) – as an anticancer platform for glioblastoma multiforme, which is an aggressive primary brain tumor (Figure 4b).^[^ ^196^ ^]^ This anticancer nanoplatform can be remotely activated under US stimulations to both release drug and generate electric stimulation. Additionally, Wang et al. utilized piezoelectric BTO nanoparticles to introduce a nondestructive, harmless, and convenient method for tooth whitening.^[^ ^197^ ^]^ This method exploits the piezo‐catalysis effect arising from using piezoelectric particles to generate ROS for bleaching tooth stains, hence whitening the pigmented teeth with minimal damage to tooth enamel and surrounding cells.

Despite their beneficial functionalities, as mentioned above, the use of nanomaterials for biomedical applications is somehow limited by the fact that only around 0.7% of the injected dose reaches the target tissue.^[^ ^198^ ^]^ Additionally, mechanisms for retention and exit at the target tissues are still poorly understood. To overcome these challenges, micro‐ and nanorobots have been proposed as these devices can swim through confined spaces in the human body by harvesting energy from different sources such as magnetic fields, US, and light.^[^ ^199^, ^200^, ^201^ ^]^ As the forces and torques on these devices can also be controlled, their residence on tissues can also be attained. These features make these devices attractive platforms as deliverers of wireless electric fields via their integration with piezo‐ or magnetoelectric materials. In this sense, researchers have developed a range of small‐scale devices to showcase the versatility of piezo‐ and magnetoelectric materials in targeted drug delivery applications.^[^ ^37^ ^]^ Pané and co‐workers introduced a soft hybrid nanorobot inspired by the electric eel (Figure 4c).^[^ ^21^ ^]^ This nanorobot features a piezoelectric tail that becomes electrically polarized when the magnetic head oscillates, generating magnetically driven motion. This mechanism enables precise control over drug release from the tail, modulated by the frequency of the applied magnetic field. Pané and co‐workers pioneered these efforts by integrating piezoelectrics and magnetoelectrics on microrobotic devices that could simultaneously transport preneuronal stem cells, and differentiate them upon awakening the polarization of the piezo‐ or magnetoelectric building blocks.^[^ ^202^ ^]^ More specifically, the authors designed a biodegradable helical micro chassis made of gelatin on which magnetoelectric core‐shell nanoparticles and progenitor neuronal cells were loaded. Upon degradation of the chassis, the nanoparticles and the cells were distributed, and the formation of a differentiated neuronal network induced by magnetoelectricity was successfully demonstrated. Jariwala et al. developed an interesting approach that utilizes piezoelectric characteristics to release drugs from a material when mechanical activation is applied.^[^ ^203^ ^]^ They created piezoelectric drug‐delivering nanofibers using electrospun PVDF‐TrFE, which generate a voltage when exposed to mechanical force. This voltage causes the surface potential to change from negative to positive, prompting the release of drugs attached to the nanofiber surface. Recently, Pané and co‐workers developed a helical magnetic microrobot decorated with piezoelectric BTO nanoparticles, enabling magnetic navigation toward a specific amyloidosis‐affected location and US‐actuated piezo‐catalytic effects to accomplish amyloid disaggregation.^[^ ^204^ ^]^ Utilizing these small‐scale untethered electric delivery machines, they have successfully demonstrated the reduction in the size of aggregated amyloid proteins by over 80% in less than 10 min by shortening and dissociating constituent amyloid fibrils under acoustic actuation.

While several examples exist in the use of piezoelectric nanomaterials for biomedical applications, a great challenge is in using biodegradable or clearable compositions (hydrodynamic diameter <5.5 nm). In the case of piezoelectric materials, especially ferroelectrics, this is a challenge, as at these small sizes, lattice contraction can lead to the partial or complete degradation of the piezoelectric properties. This would be the case with BTO, which can evolve from the noncentrosymmetric ferroelectric crystal phase to the paraelectric cubic phase.^[^ ^205^, ^206^ ^]^ Several efforts have also been made to use biodegradable piezoelectric materials such as cellulose, chitosan, collagen, silk, PLLA, for therapeutic applications.^[^ ^36^ ^]^ For example, the piezoelectricity of chitosan can be used to speed up the wound‐healing process. Recently, Chen et al. developed a chitosan‐PDA‐based composite film to accelerate wound healing by exploiting this composite system's dual functionality (i.e., piezoelectric and photothermal).^[^ ^207^ ^]^ Moreover, by performing an in vivo experiment with a rat full‐thickness wound model, they successfully validated this approach as one of the pioneering examples of combination therapy of electric stimulation and heat for wound healing. Other researchers utilized silk‐based biomaterials to heal large femoral segmented defects in nude rats^[^ ^208^ ^]^ to promote peripheral nerve regeneration^[^ ^209^ ^]^ and to develop 3D scaffolds for stem cell‐based tissue engineering applications in skeletal and connective tissues.^[^ ^210^ ^]^ Recently, Pané and co‐workers reported that modifying PLLA with 1 wt%. of high aspect ratio crystalline filler particles promotes the formation of highly crystalline and oriented piezoelectric films via drawing‐induced crystallization, hence resulting in a boosted piezoelectric stimulation performance of cells as well as oriented cell elongation along the drawing direction of the film (Figure 4d).^[^ ^211^ ^]^

A summary of the examples of current approaches in targeted therapies and small‐scale robotics is reported in **Table** **4**.

**Table 4:** Applications of organic and biodegradable piezoelectric materials in targeted therapy and small‐scale robotics.

Type of the targeted therapy	Material	Technique	Characteristics of the material/scaffold	Impact on cellular activity or disease	References
Bone regeneration	Poly (l‐lactic acid) (PLLA), and cobalt ferrite (CFO)‐bismuth ferrite (BFO) core‐shell nanoparticles	Vacuum infiltration of a PLLA/CFO‐BFO nanoparticle dispersion inside a sacrificial template composed of fused gelatin microspheres	Biodegradable 3D porous honey‐comb scaffold with magnetoelectric core‐shell nanoparticles Actuation: magnetoelectric stimulation via magnetic actuation	In vitro: 134% increase in cell (human‐derived MG63 osteoblasts) proliferation on magnetoelectrically stimulated 3D scaffolds	[189]
	Iron‐gallium (FeGa) and poly (vinylidene fluoride*‐co*‐trifluoroethylene) (PVDF‐TrFE)	DC sputtering to grow FeGa films onto Kapton and spin coating of P(VDF‐TrFE)	Flexible magnetoelectric heterostructure thin film Actuation: magnetoelectric stimulation via magnetic actuation	In vitro: magnetoelectrically promoted human osteoblast differentiation including cell proliferation, extracellular matrix (ECM) maturation, and ECM mineralization	[190]
Skin‐wound healing	Poly (l‐lactic acid) (PLLA)	Electrospinning	Biodegradable PLLA‐based piezoelectric scaffold Actuation: piezoelectric stimulation via acoustic actuation	In vitro: enhanced proliferation of fibroblast/epithelial cells, expression of genes typical for the wound healing process, and prevention against bacterial infection. In vivo: Skin regeneration in mouse models	[191]
	Chitosan and polydopamine (PDA)	Solvent casting of chitosan, and subsequent incubation in dopamine solution	Biodegradable composite film consisting of materials with piezoelectric (chitosan) and photothermal (PDA) functionalities Actuation: piezoelectric stimulation via mechanical actuation, and photothermal stimulation via near‐infrared irradiation	In vivo: in a rat full‐thickness wound model, the combination therapy enhances wound healing, with cell proliferation (keratinocytes in epidermis and fibroblasts in dermis) progresses increasingly from day 3 to day 10, through pathways mediated by heat shock protein 90 (Hsp90) and hypoxia‐inducible factor 1α (HIF‐1α)	[207]
	Poly (l‐lactic acid) (PLLA) and Barium titanate (BTO) nanoparticles	Solvent casting and hot pressing, followed by a drawing step to increase PLLA crystallization	Biodegradable, piezoelectric composite films with highly crystalline and oriented fibers Actuation: piezoelectric stimulation via acoustic actuation	In vitro: stimulation of human skin keratinocyte cells (HaCaT) enabled important cell behaviors for regeneration, e.g., cell orientation and directional migration	[211]
Neural regeneration	PVDF	Commercial poled β‐PVDF membranes	Flexible piezoelectric PVDF membrane Actuation: piezoelectric stimulation via acoustic actuation	In vitro: differentiation of neuron‐like PC12 cells upon ultrasound actuation with a performance comparable to the ones induced by neuronal growth factor	[192]
Targeted treatment of brain cancer	Poly (vinylidene fluoride*‐co*‐trifluoroethylene) (PVDF‐TrFE) nanoparticles	Lipid‐assisted nanoprecipitation of Nutlin‐3a‐loaded ApoE‐functionalized PVDF‐TrFE nanoparticles	Drug‐loaded organic piezoelectric nanoparticles to perform targeted chemotherapy and electrical treatment simultaneously Actuation: piezoelectric stimulation via acoustic actuation	In vitro: combination of chemotherapy treatment with piezoelectric stimulation resulted in activation of cell apoptosis and anti‐proliferation pathways, induction of cell necrosis, inhibition of cancer migration, and reduction of cell invasiveness in drug‐resistant Glioblastoma multiforme cell lines (T98G)	[196]
Targeted treatment of breast cancer	Poly (vinylidene fluoride*‐co*‐trifluoroethylene) (PVDF‐TrFE) and Polypyrrole (Ppy)‐nickel (Ni) nanowires	Coaxial lithography to utilize P(VDF‐TrFE) as a flexible piezoelectric tail linked to a Ppy nanowire head, decorated with Ni rings	A nanorobot based on a piezoelectric tail (P(VDF‐TrFE)) that becomes electrically polarized when the magnetic (Ni‐decorated) head oscillates, generating magnetically driven motion. Actuation: propulsion and drug release modulated via magnetic actuation	In vitro: targeted release of an anticancer drug, e.g., doxorubicin (Dox), to kill human epithelial breast cancer cells (MDA‐MB‐231). Enhanced cancer cell killing efficiency under the drug release mode, where 35% of cells were killed in comparison to 10% under the swimming mode	[21]
Targeted cell therapy for neural regeneration	Cobalt ferrite (CFO)‐bismuth ferrite (BFO) core‐shell nanoparticles and Gelatin‐methacryloyl (GelMA)	Two‐photon lithography of the photocurable GelMA‐based hydrogel chassis, loaded with magnetoelectric core‐shell nanoparticles and progenitor neuronal cells (SH‐SY5Y)	Biodegradable helical soft GelMA hydrogel chassis supports cell growth; while the magnetoelectric nanoparticles allow for the magnetic propulsion of the bioactive chassis, and act as magnetically mediated electrostimulators of neuron‐like cells Actuation: propulsion and magnetoelectric stimulation via magnetic actuation	In vitro: biodegradable helical microrobotic devices could simultaneously transport preneuronal stem cells (SH‐SY5Y) and differentiate them into neuronal networks upon awakening the polarization of the magnetoelectric building blocks	[202]
On‐demand drug release	Poly (vinylidene fluoride*‐co*‐trifluoroethylene) (PVDF‐TrFE)	Electrospinning, and loaded with drug	Piezoelectric PVDF‐TrFE membranes with alternating polarization of nanofiber surface when exposed to mechanical force, prompting the release of drug Actuation: piezoelectric actuation via mechanical stimulation	In vitro and ex vivo (porcine skin*):* controllability of drug release kinetics in a physiologically relevant environment by modulating the magnitude/dose of mechanical stimulation, which boosted the release at least of 15 times upon stimulation	[203]

## 4 Open Issues and Perspectives

A remarkable number of biological materials exhibit piezoelectric properties. Despite that, the piezoelectric properties of such natural and organic components are still far from being competitive with respect to those owned by ceramic and inorganic piezoelectric materials. Since the most common mechanical stimulus is represented by the normal out‐of‐plane stress (*d*_33_), organic materials might be more suitable for specific applications that involve the presence of in‐plane shear stresses. Otherwise, specific designs will be required to translate the normal stress into the planar one to maximize transduction efficiency. Indeed, their structure typically generated high piezoelectricity in the shear mode. As an example, glycine exhibits the shear piezoelectricity in β polymorph (*d*_16_ = 178 pC N^−1^) of 2 orders of magnitude higher than the longitudinal one described by *d*_33_ (4.7 pC N^−1^).^[^ ^212^ ^]^

Improving the organic piezoelectric material performance represents a current issue, and technological solutions to arrange the molecular chain organization, modification of material chemistry, and topological design criteria must be approached.

As already discussed in other reviews, there is still the need for a fundamental understanding and prediction of the piezoelectricity of biomaterials.^[^ ^34^ ^]^ Compared to inorganic crystals, the polarization of molecular dipoles in biomaterials is rather complicated to control. Organic materials can be organized into different polymorphs with a low crystal symmetry, and relatively low stability over time. Also, they can originate from various sources, accounting for an intrinsic inhomogeneity. For these reasons, it is fundamental to define reliable relationships among materials’ chemistry and structure, and piezoelectricity to optimize the synthesis and fabrication protocol to obtain the desired crystalline structure.

Since the polarization of crystals through electric fields is more applicable to ceramics as most biological materials are not ferroelectric, other techniques involving mechanical stretching, shear force, and capillary force (e.g., self‐assembly) should be envisaged to induce alignment in the domain orientation within the organic materials to help strengthen the piezoelectric coefficient.^[^ ^213^ ^]^ The alignment of polar domains is thought to produce more piezoelectricity. However, a random arrangement is advantageous thermodynamically, which makes randomly oriented polar domains more prevalent in nature and reduces or cancels out net polarization.^[^ ^6^, ^214^ ^]^ Alternatively, as several biomaterials possess functional groups, additional charges or dipoles can be added to the molecular assembly to increase material polarization.

Furthermore, nanoscale and microscale piezoelectricity do not always match and correspond to macroscale piezoelectricity, as the material's structure has a significant impact on piezoelectricity. Such a scalability issue implies that the same material may be strongly piezoelectric at the nanoscale but not at the microscale. This means that control of the piezoelectric behavior of that material is always necessary when varying the material size to be sure about its piezoelectricity across scales. Also, a modification of the material size implicates a modification of protocols, which may bring to an alteration of the concentration/elimination of crystal surface defects or crystal lattice contraction/expansion, leading to an increase or a decrease in the piezoelectric coefficients with respect to their macroscale counterpart. For instance, when reducing the size of the material owing to crystal lattice contraction, ferroelectric piezoelectric materials may gradually eliminate the spontaneous polarization.^[^ ^214^ ^]^ As several applications may involve the internalization of nanomaterials, and this mechanism is mainly size‐dependent, an accurate material design will be necessary to provide the piezoelectric nanomaterial the size and the internal piezoelectric structures for cell targeting and cellular uptake.

In this review, we also discuss the degradation mechanisms of these materials. The knowledge of how degradation products may interact/interfere with the physiological environment at the microscale (intra‐ or extra‐cellular level), leading to secondary effects, is a relevant aspect for a future clinical translation. Degradation environments have been resembled by physiological solutions or specific enzymes that usually are not always present in the environment of application. For example, polysaccharides (e.g., chitosan) may be principally degraded by specific enzymes (e.g., lysozyme), whose presence in the application site should be considered whenever a predetermined degradation rate is desirable. For this reason, it is always recommended that biodegradability studies be performed in environments that replicate the conditions of the application site as closely as possible to better predict the behavior of the biomaterial in vivo. Also, little knowledge still exists on how predicting the stability and durability of biodegradable piezoelectric materials, even when integrated into a composite formulation. For example, many of their polymorphs with strong piezoelectricity are metastable (e.g., β‐glycine can change its crystallinity at room temperature^[^ ^61^ ^]^). In the physiological environment, this behavior may change their inner structure over time, altering the expected performance. To minimize this effect, specific coatings for the piezoelectric biomaterials could be envisaged to reduce the direct interaction of the piezoelectric material with the external environment, slowing down the degradation rate. Also, the repetitive stimulation of such piezoelectric materials may lead to a fast decrease in performance over time, mainly due to the limited cycle lifetime, an aspect often undervalued. For example, peptides may deteriorate after continuous mechanical stimulation, potentially leading to a fast decrease of the material performance.^[^ ^212^ ^]^ To guarantee constant piezoelectric performance in vivo before their complete disintegration, the degradation rate and piezoelectricity of biodegradable piezoelectric materials must be balanced.

The next generation of organic piezoelectric materials should not only biodegrade but be resorbed by the human body to provide the stage for materials that can completely integrate into the host organism once their functionality has been thoroughly performed. However, achieving the bioresorbtion of piezoelectric biomaterials is more challenging than the sole biodegradability due to its strict requirements, which require the byproducts to be biocompatible. Bioresorbable piezoelectric nanomaterials will offer significant advantages by combining temporary mechanical and electrical functionality with natural degradation and absorption benefits.

Another point that may limit the application of a few piezoelectric biomaterials is the stiffness, which is higher if compared to human tissues and organs (e.g., collagen and FF). High piezoelectric performance always conflicts with flexibility and softness, as high piezoelectricity corresponds to high crystallinity, while flexibility and softness favor amorphousness in semicrystalline polymers.^[^ ^215^ ^]^ Piezoelectric polymers have a much lower Young's modulus than inorganic ones (e.g., ceramics), offering the stage for building more flexible composite materials. In contrast, proteins and peptides may withstand modification processes to be softened. In the alternative, playing with their size, a lower flexural rigidity may be provided to piezoelectric nanomaterials to make them more mechanically compatible for being mixed with hydrogels, for being easily injected within a liquid medium, or, in the case of microrobotics, to enhance the motile performance.

Finally, more robust protocols for piezoelectric response measurement of biomaterials should be established. The existence of different characterization methods, each affected by its artifacts, complicates the evaluation of available data in the literature, sometimes leading to large discrepancies in the comparison of material properties for the same piezoelectric nanostructures.^[^ ^216^ ^]^ Indeed, the piezoelectric coefficient for the same material can vary over some orders of magnitude, as in the case of proteins.^[^ ^6^ ^]^ This raises the question of whether the reported piezoelectric coefficients derive exclusively from the intrinsic piezoelectric properties of each material or other effects. For example, electrostriction and flexoelectricity are the two other electromechanical coupling processes that may interfere with the measurements.^[^ ^6^ ^]^ The definition of reliable and reproducible measurements of the piezoelectric properties is crucial; for this reason, standardized measurement protocols should be envisioned in the future to compare results obtained by different labs.

The use of biodegradable piezoelectric micro‐ and nanomaterials offers new prospects for all those applications where piezoelectricity plays a crucial role. In tissue engineering, for instance, US stimulation of these materials has been shown to promote several regenerative processes, such as cartilage regeneration^[^ ^217^ ^]^ or myogenesis.^[^ ^166^ ^]^ Up to now, mostly inorganic piezoelectric nanoparticles have been used for these scopes due to their remarkable piezoelectric properties.^[^ ^19^ ^]^ However, their poor biodegradability hampers the safe clinical translation of the treatment. Conversely, biodegradable piezoelectric biomaterials, though extensively studied in the form of films, nano‐ or microfibers, and composites embedded in scaffolds, as also suggested by the examples provided by this review, still face challenges in being processed into nanomaterials suitable for direct cellular delivery. Future development in this direction will hopefully provide a more direct interaction between the cells and the nanomaterials, fostering its internalization in specific cell sites. The precise localization of the nanomaterials within the cells, combined with a controlled US stimulation,^[^ ^218^, ^219^ ^]^ could significantly improve the overall therapeutic effect and might even trigger new cellular responses that have not been observed so far.

Another key aspect of the use of piezoelectric biomaterials is their precise delivery in target tissues. For specific applications, such as cancer therapies or internal wounds, it may be beneficial to incorporate these materials in actively motile structures such as micro‐ and nanorobots or use robotic dexterous catheters that could eject formulations closer to the target region.^[^ ^220^, ^221^, ^222^, ^223^, ^224^ ^]^ Biodegradable piezoelectric materials can be used in drug delivery implants, as these devices can release medication at controlled rates in response to electrical signals generated from body movements or external stimuli or be used to facilitate the blood‐brain barrier opening for the delivery of drugs into the brain.^[^ ^225^ ^]^ For example, devices made from materials like PLLA or PHB can be employed to design a device that can be activated by mechanical movements in the body or by a remote US stimulation, generating a mechanical motion or an electric field that triggers the release of the drug. In contrast, fast degradation rates should be avoided to not trigger a stronger inflammatory tissue reaction at the initial stage, as a rapid biodegradation of biomaterials elicits an acute inflammation reaction due to a significantly large production of low‐molecular‐weight compounds.

## 5 Conclusion

In this review, the potential of micro‐ and nanomaterials based on synthetic or natural biodegradable piezoelectric compounds is discussed, considering the research fields of tissue engineering, regenerative medicine, targeted therapy, and microrobotics. Thanks to their biocompatibility, biodegradability, and versatility, organic piezoelectric materials offer new opportunities in medical fields in which piezoelectricity plays a crucial role, boosting a biological response. They can potentially replace inorganic piezoelectric compounds, facilitating the clinical translation of these new therapeutic approaches. The differences in terms of materials, synthesis, and properties between inorganic (nondegradable) and biodegradable piezoelectric micro‐ and nanomaterials are described. One of the most significant challenges of biodegradable piezoelectric biomaterials is their smaller piezoelectric coefficient with respect to inorganic counterparts; techniques to improve piezoelectricity are also described, which can partly circumvent this issue. Specific examples are reported of the use of biodegradable piezoelectric biomaterials for the engineering/regeneration of different human tissues (e.g., cartilage, bone, and skeletal muscle), as well as for bringing a therapeutic effect in hard‐to‐reach regions of the human body, exploiting microrobotic platforms. Despite the current limitations to the widespread use of these organic biodegradable piezoelectric micro and nanomaterials, research on this topic is gaining momentum, paving the way to new functional and bioresorbable biomedical platforms in the near future.

## Conflict of Interest

The authors declare no conflict of interest.

## Author Contributions

**Lorenzo Vannozzi**: conceptualization (lead); data curation (supporting); project administration (supporting); resources (supporting); supervision (equal); writing—original draft (lead). **Carlotta Pucci**: data curation (equal); investigation (supporting); methodology (supporting); supervision (equal); writing—original draft (equal). **Diego Trucco**: conceptualization (supporting); data curation (supporting); investigation (supporting); supervision (supporting); writing—original draft (supporting); writing—review & editing (supporting). **Claudia Turini**: data curation (equal); formal analysis (lead); investigation (supporting); visualization (supporting); writing—original draft (supporting); writing—review & editing (supporting). **Semih Sevim**: conceptualization (supporting); investigation (equal); validation (equal); writing—review & editing (equal). **Salvador Pané**: conceptualization (supporting); funding acquisition (equal); investigation (equal); project administration (equal); resources (supporting); supervision (equal); writing—review & editing (supporting). **Leonardo Ricotti**: conceptualization (equal); funding acquisition (lead); resources (supporting); supervision (equal); visualization (supporting); writing—review & editing (lead).
